# Dynamics and Regulation of RecA Polymerization and De-Polymerization on Double-Stranded DNA

**DOI:** 10.1371/journal.pone.0066712

**Published:** 2013-06-18

**Authors:** Hongxia Fu, Shimin Le, Kalappa Muniyappa, Jie Yan

**Affiliations:** 1 Mechanobiology Institute, National University of Singapore, Singapore, Singapore; 2 Department of Physics, National University of Singapore, Singapore, Singapore; 3 Department of Biochemistry, Indian Institute of Science, Bangalore, India; 4 Centre for Bioimaging Sciences, National University of Singapore, Singapore, Singapore; Florida International University, United States of America

## Abstract

The RecA filament formed on double-stranded (ds) DNA is proposed to be a functional state analogous to that generated during the process of DNA strand exchange. RecA polymerization and de-polymerization on dsDNA is governed by multiple physiological factors. However, a comprehensive understanding of how these factors regulate the processes of polymerization and de-polymerization of RecA filament on dsDNA is still evolving. Here, we investigate the effects of temperature, pH, tensile force, and DNA ends (in particular ssDNA overhang) on the polymerization and de-polymerization dynamics of the *E. coli* RecA filament at a single-molecule level. Our results identified the optimal conditions that permitted spontaneous RecA nucleation and polymerization, as well as conditions that could maintain the stability of a preformed RecA filament. Further examination at a nano-meter spatial resolution, by stretching short DNA constructs, revealed a striking dynamic RecA polymerization and de-polymerization induced saw-tooth pattern in DNA extension fluctuation. In addition, we show that RecA does not polymerize on S-DNA, a recently identified novel base-paired elongated DNA structure that was previously proposed to be a possible binding substrate for RecA. Overall, our studies have helped to resolve several previous single-molecule studies that reported contradictory and inconsistent results on RecA nucleation, polymerization and stability. Furthermore, our findings also provide insights into the regulatory mechanisms of RecA filament formation and stability *in vivo*.

## Introduction

In *Escherichia coli*, the formation of a RecA filament on single-stranded DNA (ssDNA) is a crucial step in the processing of DSB ends during recombinational DNA repair [1]. RecA filament, formed in the presence of ATP or ATP-analogs and other co-factors, serves as an active intermediate during recombinational DNA repair [1], [2]. The formation of the RecA filament encompasses two distinct steps: a slower nucleation step and a faster polymerization step. The latter was shown to occur primarily in a 5’ to 3’ direction on ssDNA [1]–[3], while recent studies suggest that 3’ to 5’ polymerization can also occur under certain conditions [4]–[7]. RecA filament is a dynamic structure under conditions of ATP hydrolysis, which is subject to competition between polymerization and de-polymerization processes [5], [8], [9]. RecA monomer contains two distinct DNA binding sites. During RecA-catalyzed DNA strand exchange, RecA binds to ssDNA through its primary ssDNA binding site to form a nucleoprotein filament, which interacts with dsDNA weakly via its secondary site. Once sequence homology is found, RecA aligns homologous sequences, then the strands invade each other and begin the process of strand exchange [1], [10].

Although RecA primarily polymerizes on ssDNA, several studies have shown that it can also polymerize on dsDNA [8], [11]–[16]. Importantly, the RecA filament on dsDNA is proposed to be a functional state comparable to that generated during DNA strand exchange [2]. Therefore, understanding the properties of the RecA filament formed on dsDNA in physiologically relevant conditions might provide insights into the overall processes of homologous recombination.

Previously, molecular events underlying polymerization and de-polymerization of RecA on dsDNA has been investigated using single-molecule manipulation techniques [11]–[15]. Some studies have shown net RecA polymerization on dsDNA at low tensile force of several pico Newtons (pN) [11], [12], [15], while others have reported that RecA filaments were unstable at low force which resulted in net RecA de-polymerization [14]. In studies that showed net RecA polymerization at low force, an initial DNA overstretching transition at large force (∼65 pN) was often required to promote RecA nucleation [12], [14], [15], while others reported spontaneous nucleation at low force [8], [11], [13].

To date, the causes of these contradictions still remain unclear. In addition, recent experiments demonstrated that DNA overstretching, which was found to facilitate RecA polymerization on dsDNA [12], [15], in fact involves two distinct DNA structural transitions, one to ssDNA through strand-separation and the other to a non-melted elongated novel DNA structure termed as the S-DNA, whose selection can be tuned by changing base-pair stability through tuning temperature, ionic-strength, and GC-content [17]–[20]. An interesting question is whether the DNA overstretching assists in RecA polymerization through one or both of the structural transitions.

RecA polymerization involves formation of an initial nucleation site with several RecA monomers, which is followed by sequential adding new RecA monomers to the 3’ end of the filament [5]. ATP hydrolysis catalyzed RecA de-polymerization, which primarily occurs at the 5’ end, is a cooperative process and each step involves dissociation of several RecA monomers [3], [5]. As only one or a few RecA monomers are involved in each of these critical steps of RecA polymerization and de-polymerization, small DNA extension changes at the nanometer range occur. In previous single-DNA stretching studies of RecA polymerization and de-polymerization, long DNA tethers of a few microns in length were used [11]–[15]. The large longitude conformational fluctuation due to the use of long DNA prevented the previous studies from observing the detailed dynamics of competition between polymerization and de-polymerization.

Although the basis for these conflicting observations is unclear, it is possible that the use of different experimental conditions in the aforementioned studies might have contributed to the observed differences. In this paper, we systematically examined this premise at single dsDNA molecule level using magnetic tweezers. Specifically, we investigated the dynamics and regulation of the competition between RecA polymerization and de-polymerization on dsDNA by temperature, pH, salt, tensile force, dsDNA ends, and the role of DNA overstretching in single-DNA stretching experiments. The optimal conditions that permitted spontaneous RecA nucleation and polymerization, as well as conditions that could maintain the stability of a preformed RecA filament, were identified, which provided a consensus understanding of the previous contradictory experimental findings. Further, the detailed competition between polymerization and de-polymerization was monitored in real-time at a nano-meter scale.

## Materials and Methods

### DNA constructs, buffer solutions and protein

Five DNA constructs were used in our experiments: (1) 48,502 bp λ-DNA (New England Biolabs) end-labeled with a biotin and a thiol groups on the two opposite strands, (2) a 595 bp DNA with one end labeled with thiol group and the other end sealed by a short DNA hairpin labeled with biotin, (3) an 876 bp DNA that was constructed by ligating two GC rich handles to the 571 bp DNA construct (GC% = 53%). One GC-rich DNA handle was biotin labeled 153 bp DNA (GC% = 61%) and the other was thiol labeled 152 bp DNA (GC% = 65%), (4) an ∼ 600 bp DNA whose both end were sealed by short DNA hairpin labeled with biotin, (5) 3’ or 5’ tailed DNA by adding 12 nt ssDNA to the 3’ end or the 5’ end of the one-end looped 595 bp DNA construct. In experiments where 1 mM ATP was used, 1x ATP regeneration system was included to maintain ATP levels at ∼1 mM. *E. coli* RecA protein was purchased from New England BioLabs, USA or purified as previously described [21].

### Magnetic tweezers measurements

In this study a vertical magnetic tweezers setup was used to stretch the short DNA constructs. The thiol end of the DNA was covalently fixed to a sulfo-SMCC-coated (Sigma) coverglass, and the biotin end of the DNA was fixed to a streptavidin coated 2.8 –µm paramagnetic bead (Dynal M-280, Invitrogen). The bead position in the focal plane was determined by the self-correlation method at a resolution of ∼ 2 nm [22]. The bead position perpendicular to the focal plane was determined at resolution ∼2 nm by analyzing the diffraction pattern of the defocused bead image at different defocusing planes [22]. Additional details concerning the experimental set up can be found in a previously published report [23].

Forces were applied to the λ-DNA by a transverse magnetic tweezers setup [24]. The thiol end of the DNA was fixed to a sulfo-SMCC (Sigma) labeled edge of a cover glass, and the other end was labeled with biotin and was attached to a streptavidin coated 2.8 –µm paramagnetic bead (Dynal M-280, Invitrogen) to achieve high force. The DNA was placed inside a narrow flow channel so that the buffer could be conveniently replaced. A permanent magnet outside the channel applied forces from 0.01 pN up to 100 pN to the paramagnetic bead in the focal plane, and the extension of DNA was determined to be the distance from the bead to the edge of the cover glass in the force direction. The DNA was ascertained to be a single DNA tether as it underwent DNA overstretching transition at ∼ 65 pN that led to DNA elongation by ∼ 1.7 fold [25], [26].

### Temperature control

The temperature was controlled by an objective heater system (Bioptech Heater System for Olympus UPLFN 100x Objective) in the vertical magnetic tweezers or a Linkam Warm Stage Controller-MC60 in the transverse magnetic tweezers setups.

Additional details of DNA constructs, labelling, and ATP regeneration system, the force response and force calibration for short DNA tethers can be found in Supplementary Data.

## Results

### Temperature and pH switch the balance between polymerization and de-polymerization over physiological ranges

As in the previous studies, different temperature and pH values were used [8], [11]–[15], we first investigated the effects of these two factors on the polymerization of RecA on dsDNA.

**Figure 1:**
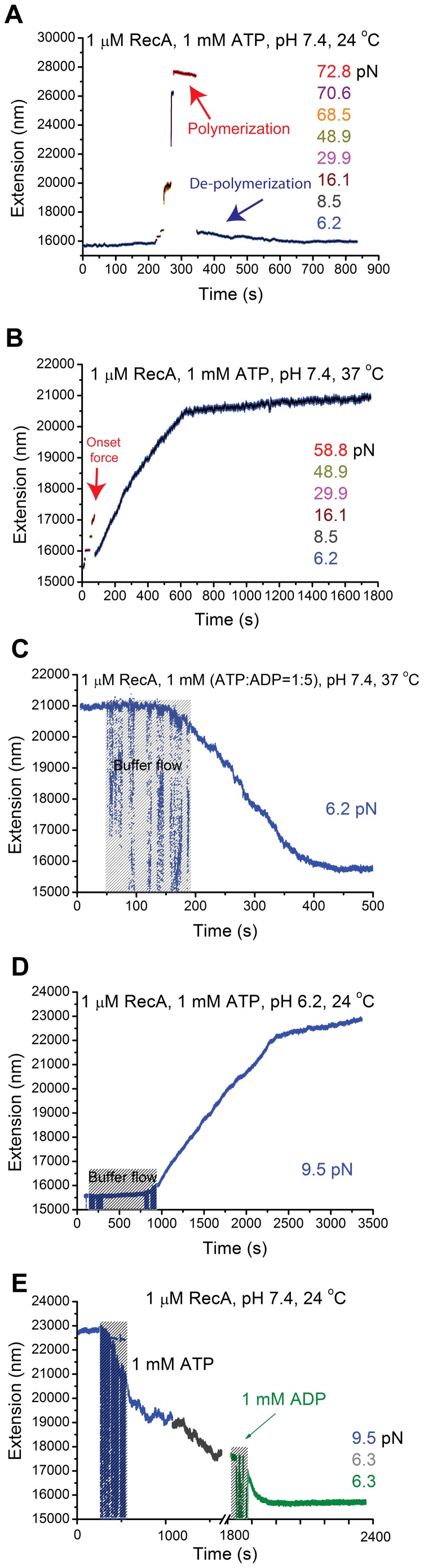
Effects of temperature and pH on the formation and stability of RecA filament. (A) Time trace of RecA polymerization and de-polymerization in a λ-DNA in 1 µM RecA, 50 mM KCl, 10 mM MgCl_2_, 1 mM ATP, 1x ATP regeneration system, pH 7.4, and 24°C, at different forces indicated by different colors. Progressive polymerization was observed at ∼72.8 pN after DNA overstretching indicated by shortening in DNA extension (red allow), while de-polymerization was observed when force was decreased to ∼ 6.2 pN (blue arrow). (B) Following the complete de-polymerization in (A), time trace was obtained on the same DNA at 37°C (other conditions remained unchanged). Progressive polymerization was observed at ∼6.2 pN (blue data points) after initiation with DNA overstretching transition by ∼58.8 pN for a short time duration (red arrow). (C) Time trace of de-polymerization of the RecA nucleoprotein filament formed in (B) after introduction of 1 mM mixture of ATP and ADP (ATP:ADP = 1∶5) (other conditions remained unchanged) at ∼ 6.2 pN. (D) Time trace of spontaneous RecA polymerization on a different λ-DNA at pH 6.2, 24°C, and 9.5 pN without initiation by DNA overstretching. (E) Time trace of de-polymerization of RecA nucleoprotein filament formed in (D) after pH was changed to 7.4 with 1 mM ATP (blue and dark grey data at different forces) and with 1 mM ADP (green data). The noisy data in (C–E) in the shadowed areas were recorded during buffer exchanging.

When we performed experiments under reaction conditions, i. e., 20 mM Tris (pH 7.4) 1 µM RecA, 50 mM KCl, 10 mM MgCl_2_, 1 mM ATP and 1x ATP regeneration system at 24°C, we observed that RecA was unable to polymerize along dsDNA over a wide range of force up to 48.9 pN, indicated by constant extensions over the respective DNA holding times at corresponding forces (Figure 1). To test whether polymerization can proceed with a pre-existing nucleation site, we applied large force of ∼ 72.8 pN to the DNA. At this force, DNA underwent an overstretching transition that led to elongation of the DNA backbone by ∼ 1.7-fold [26], as indicated in the figure panel of Figure 1A (extension at beginning of data points in red). RecA polymerization occurred at ∼ 72.8 pN, indicated by progressive shortening of DNA extension (red, Figure 1A). The shortening of DNA extension can be explained by a dynamic change from the overstretched DNA (∼ 1.7 times the B-DNA contour length [25], [26]) to the shorter extension of a fully polymerized RecA filament (∼ 1.5 times B-DNA contour length [11], [12], [14]). The extension difference between the overstretched DNA and the RecA filament is roughly (1.7–1.5)×0.34 nm/bp ≈ 0.07 nm/bp.

After holding the DNA at ∼ 72.8 pN for ∼ 80 sec, an extension reduction of ∼ 300 nm occurred, corresponding to ∼ 4,286 bp of dsDNA covered by RecA filaments. To see whether this partially polymerized RecA filament was stable and whether RecA polymerization could continue at low force, we dropped the force to ∼ 6.2 pN. Immediately after the force drop, the extension is ∼ 777 nm longer than B-DNA, confirming the existence of a large patch of partially polymerized RecA filament on the DNA. As the extension difference between the B-DNA and the RecA filament is roughly (1.5–1.0)×0.34 nm/bp ≈ 0.17 nm/bp, the result indicated that ∼ 4,571 bp of dsDNA was covered by RecA filament. The sizes of the partially polymerized RecA filament estimated at the two forces are consistent with each other within 10 percent relative error.

After the force was dropped to ∼ 6.2 pN, progressive shortening of DNA extension was observed until it returned back to the original B-DNA extension, indicating RecA de-polymerization. This result is fully consistent with the finding of Feinstein et al. [14] that pre-existing RecA filament formed on dsDNA was unstable at forces below 30 pN in similar buffer solutions. These results were confirmed by multiple independent experiments. Figure S1 shows another typical experiment which is similar to results in Figure 1A.

To examine the effect of temperature, the experiment was repeated at 37°C (other solution conditions remain unchanged). In Figure 1B, we show that, after initial RecA nucleation facilitated by the onset of DNA overstretching transition at 58.8 pN, progressive RecA polymerization occurred after force was dropped to 6.2 pN. In about 600 seconds, the extension of DNA was about 21 µm, which is ∼1.3 times the B-DNA contour length. Over the following 1,200 seconds, the extension was still growing with a much slower rate, which nearly reached a steady state. This result is consistent with the progressive growth phase and the long-lived stationary phase at low force at the same temperature reported by Shivashankar et al. [11].

Results in Figure 1A and Figure 1B together with previous studies by Shivashankar et al. [11] and Feinstein et al. [14] demonstrated that temperature is an essential factor in the regulation of the formation of RecA filament as well as its stability in the presence of ATP and magnesium. Although DNA overstretching could facilitate the initial nucleation of RecA, we note that it was not necessary at this temperature. In an independent experiment we observed spontaneous nucleation and RecA polymerization at 37°C at below 10 pN without DNA overstretching (Figure S2).

It has been reported that RecA filaments experience dynamic instability during ATP-hydrolysis, caused by the unstable state when RecA is associated with ADP [1], [3]. Utilizing this property, we washed RecA bound on the same dsDNA tether using solution containing 1 µM RecA, 50 mM KCl, 10 mM MgCl_2_, 1 mM (ATP:ADP = 1∶5) at the same temperature. As shown in Figure 1C, after the solution was introduced, net disassembly of the RecA filament started in a stepwise manner until DNA completely returned to the B-DNA state within ∼ 500 sec, which allowed to reuse the DNA for further investigations.

In addition to temperature, different pH values were also used in a number of previous studies; therefore, we examined the effect of pH on the formation of RecA filament and stability at 24°C. In Figure 1D, in reaction conditions containing 20 mM MES (pH 6.2), 1 µM RecA, 50 mM KCl, 10 mM MgCl_2_, 1 mM ATP, 1x ATP regeneration system, we show that spontaneous RecA nucleation and polymerization started while the protein was introduced without assistance of DNA overstretching transition. However, when pH was raised to 7.4, the nearly fully polymerized RecA filament was rapidly de-polymerized (Figure 1E). Similarly, Figure S3 shows spontaneous nucleation and filament formation at below 10 pN under the same experimental condition as those used in Figure 1D.

The results depicted in Figure 1A–E demonstrate that RecA filament formation and stability on dsDNA is regulated by temperature and pH. At higher temperature and lower pH values, RecA filament is stable and can polymerize at low force, while at lower temperature and higher pH, RecA filament is unstable at low force resulting in net de-polymerization of pre-existing RecA filaments. Under conditions when net RecA polymerization was favored, initial nucleation could be greatly facilitated, although not absolutely necessary, by DNA overstretching transition.

### Detailed dynamics of RecA polymerization and de-polymerization

The data presented in the previous section have shown that temperature and pH as well as force can fine-tune the balance between RecA polymerization and de-polymerization on dsDNA, which led to an increase in DNA extension in the polymerization phase and decrease in the de-polymerization phase at below DNA overstretching transition force. Next, we sought to examine the competition dynamics using short 595 bp DNA that significantly improves signal-to-noise ratio by suppressing the longitude fluctuation of DNA [23]. Due to the use of the short DNA tether and re-orientation of bead during force change, the absolute DNA extension and the extension change at different forces cannot be accurately determined. However, at the same force, the bead orientation is fixed and the extension change at the constant forces can be accurately measured (Methods S1). Therefore, on short DNA tethers we only focus on extension changes at constant forces.

**Figure 2:**
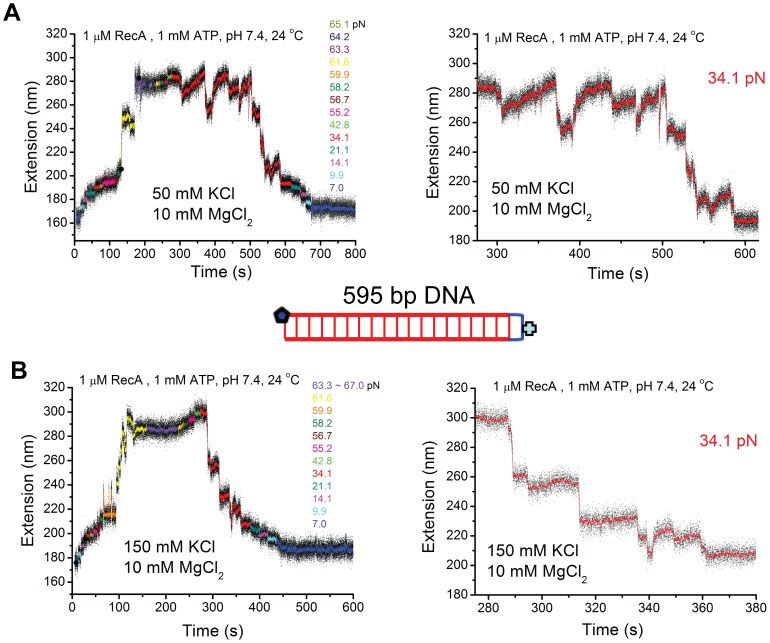
Detailed dynamics of short RecA filaments in different KCl concentrations. (A–B) Time traces of polymerization and de-polymerization of RecA nucleoprotein filament on a 595 bp dsDNA in 1 µM RecA 1 mM ATP, 1x ATP regeneration system, 24°C, pH 7.4, with 50 mM KCl (A) first then 150 mM KCl (B) next. Data in the left panels were recorded at different forces indicated by different colors. Right panels show dynamics of the competition between polymerization and de-polymerization under a constant force of ∼34.1 pN. Inset shows the sketch of the 595 bp DNA containing one closed end and one open end.

The data in Figure 2A–B were generated using the same 595 bp DNA tether in buffered solutions containing 10 mM MgCl_2_, 20 mM Tris-HCl (pH 7.4), 1 uM RecA, 1 mM ATP, 1x ATP regeneration system, and either 50 mM or 150 mM KCl, at 24°C, respectively. As RecA filament is unstable at low force 24°C; DNA overstretching transition was provided to abet RecA polymerization. As one end of the DNA is topologically closed, potential complete strand-dissociation during DNA overstretching was avoided. After RecA polymerization nearly completed, the force was decreased and the DNA extension dynamics was recorded at constant forces over long time.

Figure 2A, left panel shows the time course of the experiment from overstretching assisted polymerization to de-polymerization at lower force values. RecA was fully polymerized at force greater than 63.3 pN. The right panel shows the extension dynamics at a constant force of ∼34.1 pN over ∼ 380 seconds. Interestingly, we observed saw-tooth dynamics pattern, indicated by stochastic abrupt extension drops with various sizes up to ∼ 30 nm and very slow extension elongation processes between successive extension drops. At this force, the de-polymerization (abrupt extension drops) only slightly out-competed the polymerization (slow extension elongation processes). We note that the kinetics of decrease in DNA extension from ∼ 280 nm to ∼ 190 nm required an extended time period of > 300 seconds. At lower force values, further net RecA de-polymerization occurred and the DNA extension returned to the B-DNA extension after the de-polymerization was completed.

To examine whether the saw-tooth dynamics pattern is sensitive to salt concentration, we increased the KCl concentration to 150 mM (the other factors remained unchanged) and repeated the experiments with the same DNA (Figure 2B). Similar to Figure 2A, the polymerization was assisted by the onset DNA overstretching transition force of ∼61.6 pN. The force was subsequently reduced to 34.1 pN. Consistent with the behavior in the presence of 50 mM KCl, we observed dynamic de-polymerization of RecA, as indicated by stochastic abrupt extension drops with various sizes up to ∼ 30 nm. However, apparent re-polymerization events after de-polymerization events were much less often. After each de-polymerization step, DNA extension often remained at a plateau until the next de-polymerization event. Due to the significantly reduced re-polymerization activity in 150 mM KCl compared to that in 50 mM KCl, the de-polymerization process predominated, resulting in much faster net de-polymerization (required only 100 seconds to drop from ∼280 nm to ∼ 200 nm). Figure S4(A-B) shows another typical independent experiment that demonstrated consistent effects of KCl concentration on the stability of RecA nucleoprotein filament formed on dsDNA.

**Figure 3:**
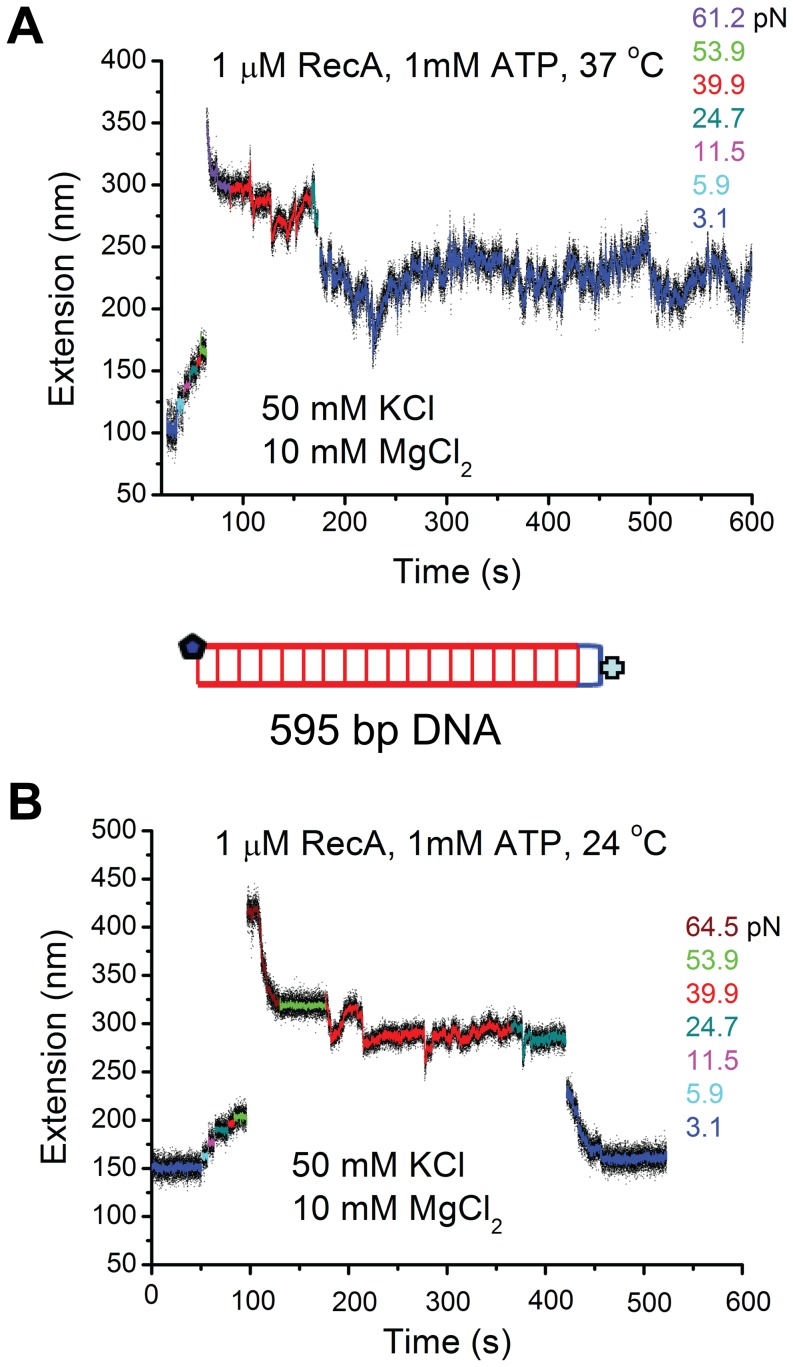
Effects of temperature on short RecA filaments. (A–B) Time traces of polymerization and de-polymerization of RecA nucleoprotein filament on another 595 bp dsDNA tether in 1 µM RecA 1 mM ATP, 1x ATP regeneration system, 50 mM KCl, 10 mM MgCl_2_, pH 7.4, at 37°C (A) first then 24°C (B) next. Different colors indicate data at different forces. The results revealed stable RecA nucleoprotein filament at 37°C and unstable RecA nucleoprotein filament at 24°C at low force, consistent with results obtained on large λ-DNA (Figure 2A–B).

Next, we re-investigated the effects of temperature using short DNA. Figure 3A shows the extension time course of fully polymerized RecA filament at different decreased forces in 50 mM KCl, 10 mM MgCl_2_, at 37°C. At ∼ 40 pN, saw-tooth extension dynamics was observed but without apparent net de-polymerization over the observation time period. Further, after we reduced the force to a much lower value of ∼ 3 pN, saw-tooth dynamic fluctuation still remained without net de-polymerization. The average extension was ∼100 nm longer than the B-DNA extension, corresponding to ∼50% DNA elongation suggesting a fully polymerized RecA filament.

After removal of RecA by 5 mM ADP, the experiment was repeated at 24°C using the same DNA at 24°C. Like in Figure 2A, we observed saw-tooth dynamic extension fluctuation at ∼ 40 pN (Figure 3B). However, at lower forces, net de-polymerization occurred. Consistent with the observations shown in Figure 1A and Figure 2A, rapid net de-polymerization manifested at ∼ 3 pN until the DNA extension returned to the B-DNA extension. Effects of pH were also re-examined using short DNA tethers at 24°C, which revealed stable RecA polymerization at < 10 pN in pH 6.1 (Figure S5).

The results obtained from stretching short DNA shown in Figures 2–3 and Figure S5 consistently demonstrated that formation of stable RecA filament at low force is favored at 37°C or pH 6.1, which becomes unstable when temperature was decreased to 24°C at pH 7.4. In addition, due to the significantly increased signal-to-noise ratio by stretching short DNA tethers, a novel dynamic saw-tooth pattern in DNA extension time traces was observed. This saw-tooth pattern clearly indicates that de-polymerization of RecA in a dsDNA is a highly stochastic abrupt process involving stepwise extension reduction in a wide range roughly 5 – 40 nm, indicating a highly cooperative de-polymerization process involving large patches of RecA filament covering 10 – 80 bp DNA segments. In contrast to the highly stochastic cooperative de-polymerization process, the polymerization is a much slower progressive process that does not involve clear steps that could be determined by our instrument.

### Single-stranded DNA produced during the DNA overstretching transition facilitates RecA polymerization on dsDNA

Many previous studies of RecA polymerization on dsDNA utilized DNA overstretching transition at ∼ 65 pN, which leads to DNA elongation by ∼ 1.7-fold, to assist RecA nucleation and polymerization. Yet, the mechanism of how DNA overstretching facilitates the nucleation step remains unclear. Recent studies have demonstrated that, torsion-unconstrained DNA undergoes three different structural re-organizations during DNA overstretching: (i) “peeling” apart of dsDNA to produce a peeled-ssDNA strand under tension while the other strand coils, (ii) “inside-strand-separation” of dsDNA to two parallel ssDNA strands that share tension (melting bubble), and (iii) “B-to-S” transition to a novel base paired dsDNA, termed “S-DNA” [17]–[20], [27]. Among the three transitions, the strand peeling and the B-to-S transition are predominant in physiological solution conditions at similar force of ∼ 65 pN, which can co-exist under certain solution conditions [17]–[20], [27]. The selection of the B-to-S transition and the strand-peeling transition depends on factors that change DNA base pair stability [17]–[20], [27]. At higher base pair stability (high salt concentration, low temperature, high GC percentage), the B-to-S transition is selected over the strand-peeling transition [17]–[20], [27]. The question waiting to be addressed is whether the ssDNA produced in strand peeling transition or the S-DNA produced in B-to-S transition assisted RecA nucleation.

RecA is known to bind ssDNA with high affinity [1]. Therefore, one intrinsic mechanism would be that ssDNA produced through strand-peeling transition or the inside-strand separation promotes RecA nucleation on the released ssDNA and polymerize along ssDNA into the dsDNA region. For DNA with open ends (the DNA used here) or with nicks, the strand-peeling transition predominates compared to the inside-strand separation [27], [28], so one only needs to consider the role of strand-peeling transition in DNA overstretching assisted RecA polymerization under our experimental conditions. It should be noted that under our experimental conditions in Figures 1–3, significant amount of ssDNA was produced through the strand-peeling transition, for both the λ DNA and the 595 bp DNA (Figure S6).

**Figure 4:**
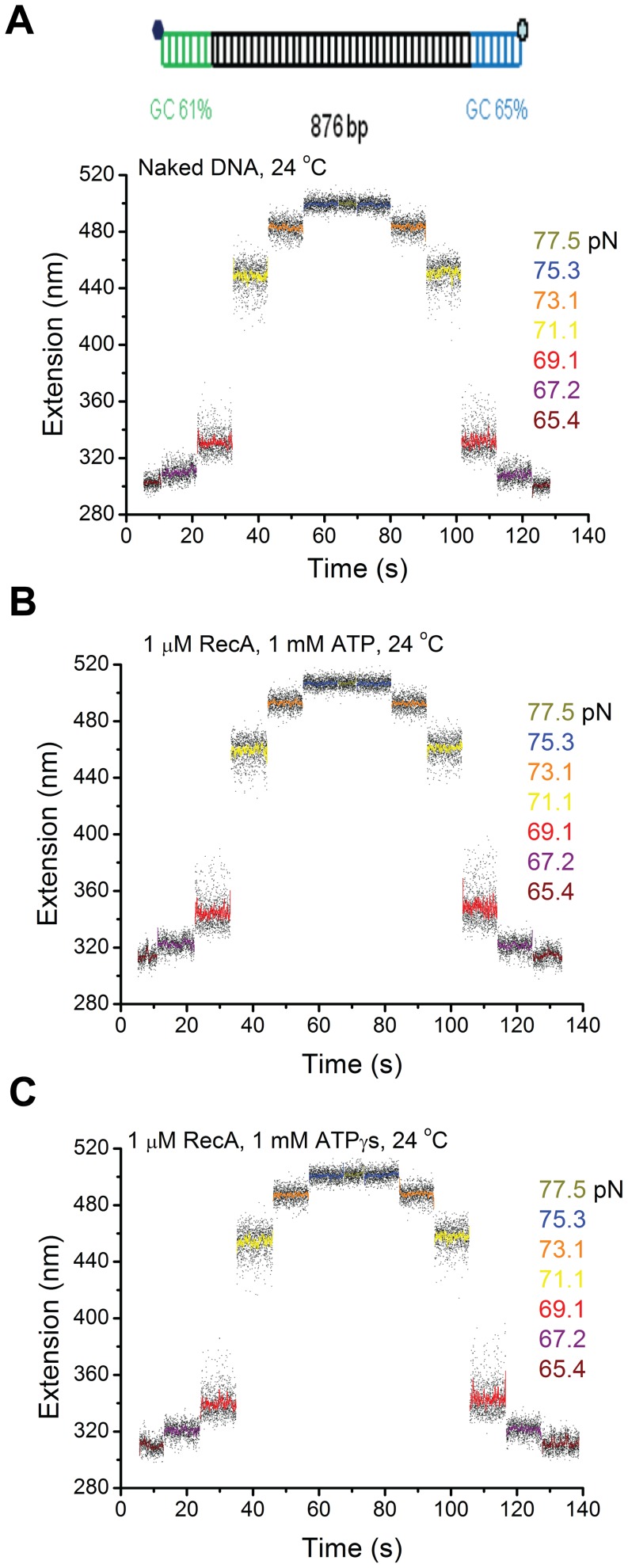
The S-DNA produced in the B-to-S transition does not promote RecA nucleoprotein filament formation. (A) Time traces of the extension of an 876 bp DNA tether with two GC-rich ends recorded during B-to-S transition in force-increase scan and the reverse S-to-B transition in the subsequent force-decrease scan in 50 mM KCl, 10 mM MgCl_2_, pH 7.4, at 24°C. The transitions are completely reversible, indicated by the same extensions recorded at the same forces during the force-increase and force-decrease scans. Inset on the top shows a sketch of the 876 bp DNA; (B–C) Results identical to those in (A) were obtained when the same experiments were repeated on the same DNA tether in 1 µM RecA and 1 mM ATP, 1x ATP regeneration system (B) or in 1 µM RecA and 1 mM ATPγS (C), indicating no RecA filaments formed on the S-DNA produced by the B-to-S transition within the experimental time scale.

An interesting alternative possibility would be that the novel double-stranded S-DNA produced through B-to-S transition is the actual substrate for RecA, which was proposed based on the similarly elongated DNA backbones between the S-DNA and fully polymerized RecA filaments [13], [18], [20]. To further test this possibility, we investigated the effect of pure B-to-S transition on the RecA polymerization using an 876 bp DNA construct which contained two short GC-rich handles to the two ends of DNA (Figure 4A). These two GC-rich handles prevented strand peeling from the two DNA ends, and the resulting DNA overstretching transition became a pure B-to-S transition, as demonstrated in our previous paper [18].

Figure 4A shows force-response of DNA with GC-rich handles under the assay conditions in the absence of RecA. The transition, indicated by increased DNA extension variance, started at ∼ 65 pN and finished at ∼ 75 pN. It is a pure B-to-S transition indicated by the lack of hysteresis between the extensions recorded in the force-increase scan and in the force-decrease scan [17]–[20] (Methods S1). Figure 4B & C show the DNA extension time courses recorded on the same DNA in the presence of 1 µM RecA with ATP (B) or ATPγS (C) as co-factors. The results were identical to Figure 4A, indicating the absence of RecA nucleation and polymerization in the overstretched DNA. Figure S7A–B show another typical independent experiment that consistently demonstrated that the RecA is unable to nucleate and polymerize on S-DNA.

Taken together the results in Figure 2A, Figure 3B, Figure 4B and Figure S7, which were obtained under identical solution conditions, we conclude that the DNA overstretching transition assisted RecA polymerization, which always started within seconds after DNA overstretching, occurred mainly through binding to ssDNA produced by the strand-peeling transition. In other words, S-DNA is not a preferable binding substrate for RecA filament formation.

**Figure 5:**
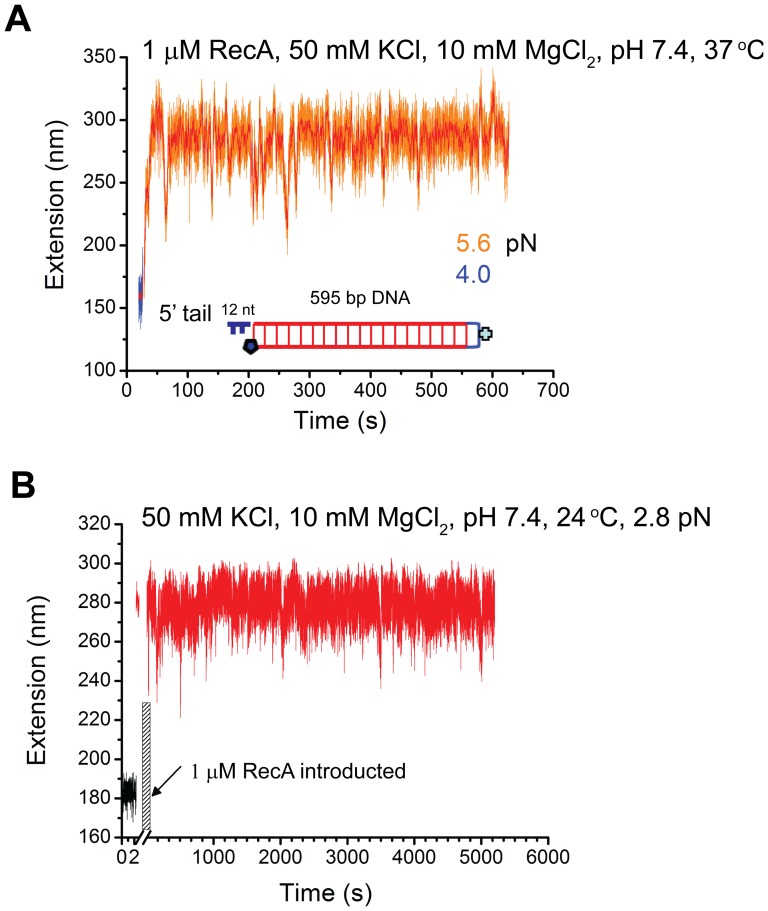
Effects of ssDNA overhangs on the formation of RecA nucleoprotein filaments. (A) Time traces of the extension of one end-closed 595 bp DNA with a 12 nt 5’ ssDNA tail in 1 µM RecA, 1 mM ATP, 1x ATP regeneration system, 50 mM KCl, 10 mM MgCl_2_, pHpH 7.4, at 37°C. A stable RecA nucleoprotein filament was formed after a spontaneous RecA polymerization at around 5 pN. (B) Time traces of a DNA with the same structure as the DNA in (A) at 24°C (other conditions remained unchanged). A stable RecA nucleoprotein filament was also observed for over 5000 second, after a spontaneous RecA polymerization at around 3 pN. The shadowed area represents the process of solution flow with RecA.

The foregoing conclusion predicts that, under solution conditions that favor net RecA polymerization, a pre-existing ssDNA overhang should also facilitate initial RecA nucleation without DNA overstretching transition. Several lines of evidence have established that polymerization of RecA proceeds in the 5’- 3’ direction, and requires a minimal of ssDNA of ∼9 nucleotide residues (for binding of three RecA monomers) for initial nucleation [8]. Therefore, we tested the prediction using a 595 bp DNA construct containing a 5’ ssDNA overhang of 12-nt. Figure 5A shows a time-course of DNA extension in solution containing 1 µM RecA, 1 mM ATP, 1x ATP regeneration system in 50 mM KCl, 10 mM MgCl_2_, 20 mM Tris (pH 7.4) at 37°C. We found that rapid RecA polymerization occurred spontaneously when the RecA solution was introduced to channel at ∼ 5.6 pN, and reached a steady state in only ∼ 40 seconds. The DNA became ∼120 nm longer (∼50% to B-DNA) than that before the RecA solution was introduced, indicating a fully formed RecA filament. The DNA extension underwent quick and large dynamic fluctuation, but the average extension remained constant over a time period of ∼ 600 seconds. As a control, the same experiment was repeated with another DNA construct containing a 3’ ssDNA overhang of 12-nt ligated to the 576 bp dsDNA (other experimental conditions remain unchanged). Over the time period of ∼1500 seconds, spontaneous RecA polymerization did not occur on the DNA at similar force ∼ 6.4 pN (Figure S8A). In addition, the extension fluctuation is much less dynamic (smoother and smaller variance) compared to Figure 5A; the standard variance of the extensions (orange data) in Figure 5A&B are 18 nm and 4 nm, respectively. When the force was subsequently increased to > 60 pN where DNA overstretching transition began, RecA polymerization immediately started and the resulting RecA filament was stable (Figure S8B).

Figure 5A demonstrated that DNA with pre-existing 5’ overhang at 37°C could dramatically facilitate RecA nucleation compared to the same DNA sequence without the 5’ overhang. This is consistent with the previous conclusion that the DNA overstretching assisted RecA polymerization is mainly through the ssDNA produced during the transition. The results further establish that the RecA polymerization in a dsDNA is mainly through polymerization on ssDNA strand in a 5’ to 3’ direction.

Interestingly, similar rapid spontaneous RecA nucleation and polymerization on the one-end-closed 596 bp DNA with a 12 nt 5’tail overhang was also observed at 24°C in solution containing 1 µM RecA, 1 mM ATP, 1x ATP regeneration system in 50 mM KCl, 10 mM MgCl_2_, 20 mM Tris (pH 7.4) (Figure 5B). The resulting stable RecA filament was ∼ 110 nm longer (∼ 50%) than B-DNA for a force range of 2–40 pN (Figure S9). The DNA extension also underwent a quick and large dynamic fluctuation, but the average extension remained constant over a time period of ∼5000 seconds (Figure 5B). Figure S10A–B show two other typical independent experiments where spontaneous RecA polymerization and resulting stable RecA filaments over > 1000 second were observed at several pN forces at 24°C. These results are contrast to previous results obtained on DNA with blunt ends (Figure 1A, Figure 2A, and Figure 3B as well as Figure S4A), where pre-formed RecA filaments in the same condition are unstable and undergo step-wise de-polymerization. This results highlight that the ends of DNA have a major role in regulating both the RecA nucleation and the overall stability of a preformed RecA filament. In the Discussion section, we propose an end-capping mechanism to explain the stable RecA filament in 24°C and pH 7.4 on DNA with 5’ overhang.

## Discussion

In this study, we investigated the effects of multiple physiological factors including temperature, pH, ionic strength, and tensile force, as well as DNA ends that govern RecA polymerization on dsDNA at single molecule level. We found that the competition between RecA polymerization and de-polymerization is exquisitely regulated by solution conditions over a wide range of physiological changes (24 – 37°C, pH 6.2 – 7.4, 50 – 150 mM KCl) and the ends of DNA. The effects of the three most critical factors (temperature, pH, and the DNA ends) are summarized in Table 1.

**Table 1:** Summary of main results of RecA nucleation, polymerization, and stability.

Experimental Conditions a	RecA nucleation, polymerization, and stability
24°C, pH 7.4; DNA with blunt ends (48,502 bp λ-DNA; 595 bp one-end-sealed DNA)	1. Nucleation requires force-induced DNA strand-peeling transition; 2. Polymerization requires high force (> 40 pN); 3. Pre-formed RecA filament is unstable at forces of several pN;
24°C, pH 7.4; DNA with 5’ ssDNA tail (595 bp DNA with 12 nt 5’ ssDNA tail)	1. Spontaneous nucleation and polymerization without assistance of DNA strand-peeling; 2. Pre-formed RecA filament is stable at forces of several pN;
24°C, pH 7.4; 876 bp DNA with two GC rich handles, 600 bp GC rich end-closed DNA	Nucleation and polymerization do not occur during DNA B-to-S transition;
37°C, pH 7.4; DNA with blunt ends (48,502 bp λ-DNA; 595 bp one-end-sealed DNA)	1. Nucleation requires force-induced DNA strand-peeling transition; 2. Progressive polymerization occurs at forces of several pN; 3. Pre-formed RecA filament is stable at forces of several pN;
37°C, pH 7.4; DNA with 5’ ssDNA tail (595 bp DNA with 12 nt 5’ ssDNA tail)	1. Spontaneous nucleation and polymerization without assistance of DNA strand-peeling; 2. Pre-formed RecA filament is stable at forces of several pN;
24°C, pH 6.2; DNA with blunt ends (48,502 bp λ-DNA; 595 bp one-end-sealed DNA)	1. Spontaneous nucleation and polymerization occur at forces of several pN; 2. Pre-formed RecA filament is stable at forces of several pN;

a All experiments include 1 µM RecA, 10 mM MgCl_2_, 50–150 mM, 1 mM ATP and 1X ATP regeneration system.

Here, we reconcile a large number of apparently discrepant results. An initial study reported spontaneous RecA polymerization on λ DNA at low force (∼ 6 pN), high temperature (∼ 37°C) and low pH (∼ pH 6.8) [11]. The spontaneous RecA polymerization unaided by DNA overstretching can be explained by the fortuitous presence of 5’ ssDNA overhangs. Similarly, others have reported net RecA polymerization on dsDNA at both high (37°C) and low temperature (22°C), at low pH (∼ pH 6.2) and low force [8], [29]. These findings are consistent with ours in the similar experimental conditions. However, another study reported net RecA polymerization on dsDNA only at large force [14]. At forces less than 30 pN, pre-formed RecA filaments were found to be unstable and rapidly dissociated from DNA. As their experiments were conducted at low temperature (∼25°C), high pH (∼ pH 7.5), these results are consistent with our results obtained under similar conditions.

At pH 7.4 in 24°C – 37°C, for dsDNA with blunt ends, nucleation requires overcoming a large kinetic barrier, which can be relieved by ssDNA produced through DNA overstretching. For a partially formed RecA filament, its fate is determined by a competition between progressive polymerization and abrupt de-polymerization events. At 37°C, polymerization outcompetes de-polymerization, resulting in a fully polymerized stable RecA filament at forces of several pN. In contrast, at 24°C and force below ∼ 40 pN, de-polymerization outcompetes polymerization, resulting in a saw-tooth dynamics in DNA extension with overall stepwise net de-polymerization.

**Figure 6:**
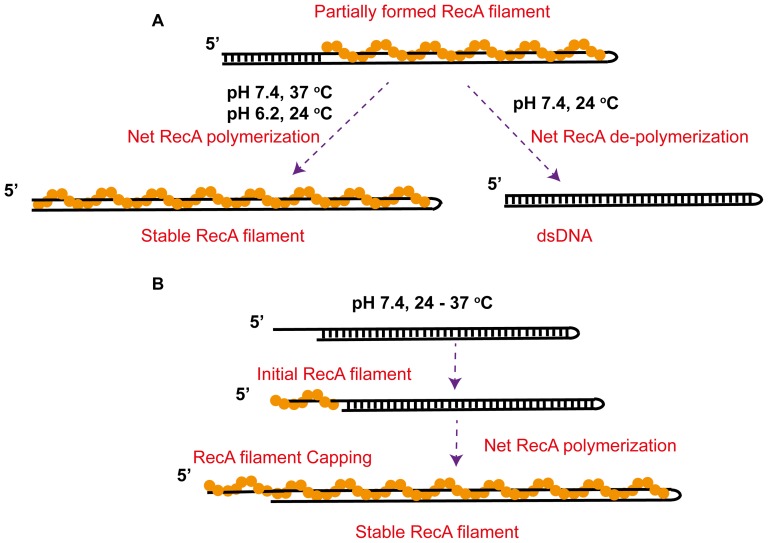
Mechanistic models of the stability of RecA filaments formed on dsDNA. (A) On dsDNA with blunt ends, at 37°C and pH 7.4 or 24°C and pH 6.2, polymerization of a partially formed RecA filament outcompetes DNA re-hybridization, leading to a net extension of the RecA filament into a stable fully coated RecA filament. In contrast, at 24°C and pH 7.4, DNA re-hybridization outcompetes RecA polymerization, leading to a net de-polymerization of the RecA filament into a stable B-form DNA. (A) On dsDNA with a 5’ ssDNA overhang that provides sites to initial RecA nucleation and polymerization, invasion of the RecA filament formed on the ssDNA overhang into the dsDNA region occurred in 24–37°C and pH 7.4, leading to stable fully coated RecA filament explained by the end-capping mechanism discussed in the text.

It has been known that RecA polymerizes from 5' to 3' on ssDNA, whereas RecA de-polymerization primarily occurs from the 5' end during ATP-hydrolysis[1]–[3]. At the 5' end of the ssDNA strand that is polymerized with RecA, RecA dissociation may occur during ATP-hydrolysis. Then, the vacated ssDNA hybridizes with the complimentary strand, resulting in formation of base paired dsDNA at the 5’ end that prevents rebinding of RecA in this region of DNA (Figure 6A). This mechanism explains the abrupt stepwise de-polymerization events observed in 24°C. The slow re-polymerization process at 24°C likely indicates a reversed RecA polymerization process from 3’ to 5’ that was reported recently [4]–[7]. Higher force or higher temperature may increase the RecA filament stability and decrease the DNA base pair stability, which leads to net progressive polymerization and formation of stable RecA filaments.

In contrast, in the same conditions (pH 7.4, 24°C – 37°C), for DNA with a 12 nt 5’ ssDNA overhang, spontaneous RecA nucleation and polymerization occur at forces of several pN, resulting formation of a stable filament. Compared to the unstable RecA filaments formed on DNA with blunt ends at 24°C, this result indicates that the ssDNA tail regulates the stability of the entire filament. We propose a 5’ end-capping mechanism to explain the results. A 5’ ssDNA overhang provides a nucleation site, which allow RecA polymerization invading into the dsDNA region. On DNA polymerized with a RecA filament, RecA dissociation still occurs at the 5’ end of the ssDNA overhang during ATP hydrolysis. However, unlike the case of DNA with blunt ends, there is no complementary strand to re-hybridize with the transiently vacated ssDNA sites; hence, the vacated DNA will be re-occupied by RecA re-polymerization resulting in stabilizing the entire RecA filament (Figure 6B).

At pH 7.4, for dsDNA with blunt ends, overcoming a large kinetic barrier is necessary, which can be relieved by ssDNA produced through DNA overstretching or by adding a 5’ ssDNA overhang to the dsDNA. In contrast, at pH ∼ 6.2, spontaneous RecA polymerization on DNA with blunt ends always occurred at low force immediately after RecA solution was introduced, suggesting that at low pH the energy barrier for initial strand invasion is significantly reduced, either from the ends or inside the DNA.

The complex regulation of RecA polymerization on dsDNA may depend on many other factors and their combinations including concentrations of nucleotides, RecA, MgCl_2_, the type of nucleotide (ATP, ATPγS, and ADP, etc) [10]. In particular, it was shown that RecA polymerization at moderate forces lead to dsDNA unwinding [15], [30]. Also, RecA from different bacterial species may have different kinetics and stability when they form filaments on dsDNA. These additional factors will be investigated in our future studies.

Previous experiments employed DNA overstretching to promote RecA nucleation and for subsequent RecA polymerization on dsDNA. Our results show that ssDNA produced through strand-separation transition during DNA overstretching is the main factor that alleviates the nucleation barrier. Consistently, we found that the elongated base paired S-DNA through the B-to-S transition during DNA overstretching did not promote initiation of RecA polymerization, contrary to previously proposed possibility that the S-DNA might be a binding substrate of RecA [13], [17], [18], [26].

During RecA-catalyzed DNA strand exchange, RecA binds to ssDNA through its primary DNA binding site and forms a nucleoprotein filament [1]. Interestingly, the secondary DNA binding site can also bind ssDNA with a much higher affinity than to dsDNA [31]. Our results suggest that RecA polymerization on dsDNA occurs likely via ssDNA in a 5’-3’ direction and the displaced strand from dsDNA is held by the secondary DNA binding site. This culminates in the formation ssDNA-RecA-ssDNA co-filament, consistent with the model proposed by Pugh and Cox [10].

In summary, our findings provide a greater understanding of how temperature, pH, DNA overstretching, and ssDNA overhangs affect RecA function and dynamics. Additionally, we believe that the data presented here help reconcile conflicting results of single**-**molecule studies in regard to RecA polymerization and RecA filament stability on dsDNA. Finally, as RecA filament formed on dsDNA is believed to represent a functional state during DNA strand exchange, these results may also provide insights to the RecA activities *in vivo*.

## Supporting Information

Figure S1 **Time trace of RecA polymerization and de-polymerization on a λ-DNA in 1 µM RecA, 50 mM KCl, 10 mM MgCl_2_, 1 mM ATP, 1X ATP regeneration system, pH 7.4, and 24 °C at different forces indicated by different colors.** Progressive polymerization was observed at ∼73 pN after DNA overstretching indicated by shortening in DNA extension (Red), while de-polymerization was observed when force was decreased to ∼5.6 pN (black).
(PNG)

Figure S2 **Time trace of spontaneous RecA polymerizing on a λ-DNA in 1 µM RecA, 50 mM KCl, 10 mM MgCl_2_, 1 mM ATP, 1X ATP regeneration system, pH 7.4, and 37°C, at a force of ∼ 7 pN.** Progressive polymerization was observed (blue arrow) before the DNA was broken after 800 second (orange arrow). The noisy data in the shadowed areas were recorded during buffer exchanging.
(PNG)

Figure S3 **Time trace of spontaneous RecA polymerizing on a λ-DNA in 1 µM RecA, 50 mM KCl, 10 mM MgCl_2_, 1 mM ATP, 1X ATP regeneration system, pH 6.2, and 24°C, at a force of ∼ 3.1 pN.** The noisy data in the shadowed areas were recorded during buffer exchanging.
(PNG)

Figure S4 **Effects of KCl concentration on the dynamics of RecA filaments.** (A–B) Time traces of polymerization and de-polymerization of RecA filament on a 595 bp dsDNA in 1 µM RecA, 10 mM MgCl_2_, 1 mM ATP, 1X ATP regeneration system, pH 7.4, and 24°C, with 50 mM KCl (A) first then 150 mM KCl (B). In both A and B, progressive RecA polymerization was observed after the force was jumped from 44.1 pN to 67.1 pN (red). Different dynamics of de-polymerization and polymerization of 50 mM KCl and 150 mM KCl were observed when force was jumped back to 44.1 pN (blue).
(PNG)

Figure S5 **Time trace of spontaneous RecA polymerizing on a 595 bp DNA in 1 µM RecA, 50 mM KCl, 10 mM MgCl_2_, 1 mM ATP, 1X ATP regeneration system, pH 6.1, and 24°C, at a force of ∼10 pN and stable RecA filament at < 3 pN.** The noisy data in the shadowed areas were recorded during buffer exchanging.
(PNG)

Figure S6 **Extension hysteresis between force-decrease scan and force increase scan during the strand peeling transition.** Time trace of force-increase scan and force-decrease scan of a 595 bp DNA in 50 mM KCl, 10 mM MgCl_2_, 1 mM ATP, 1X ATP regeneration system, pH 7.4, and 24°C, at different forces indicated by different colors. The hysteresis indicated by different extensions at 53.9 pN (orange) between the two force-scans suggests that the DNA went through a strand peeling transition during the force-increase scan.
(PNG)

Figure S7 **Time traces of an ∼600 bp two-ends-closed DNA in 50 mM KCl, 10 mM MgCl_2_, pH 7.4, and 24°C, without RecA (A) or with 1 µM RecA,1 mM ATP, 1x ATP regeneration system (B).** The DNA overstretching transitions in both A and B are completely reversible and without hysteresis, indicated by the same extensions recorded at the same forces during the force-increase and force-decrease scans. These results indicate that no RecA filaments formed on the S-DNA produced during B-to-S transition within the experimental time scale. Inset shows a sketch of the end-closed DNA.
(PNG)

Figure S8 **Time traces of the extension of a 595 bp DNA with a 12 nt 3' ssDNA tail and another end sealed.** (A) Time traces of the extension of the DNA in 1 µM RecA, 50 mM KCl, 10 mM MgCl_2_, 1 mM ATP, 1x ATP regeneration system, pH 7.4, and 37°C. Within the experimental time scale of 1600 seconds, the DNA extension remained at the B-DNA extension, indicating that 3' ssDNA overhang did not promote RecA filament formation at low force. (B) Time trace of the same DNA in the same solution and temperature condition, when the force was subsequently increased to > 60 pN where DNA overstretching transition occurred, the RecA polymerization immediately started and the resulting RecA filament was stable. When the force was reduced to 6.4 pN, the DNA extension was still ∼120 nm longer than the B-DNA before RecA polymerization, indicating a stable RecA filament at low force.
(PNG)

Figure S9 **Force responses of a 595 bp one-end closed DNA with a 12 nt 5' ssDNA (Black), and the same DNA with RecA filament formed (Red).** The extension of the DNA formed with RecA filament is about 50% longer than that of naked DNA before RecA was introduced. After remove the RecA by exchanging to pure buffered solution, the RecA filament de-polymerized and resulting DNA extension (blue) overlaps with naked DNA extension. The DNA used in this experiment is the same as that used in Figure 5B in the main text.
(PNG)

Figure S10 **Spontaneous RecA polymerization on two independent 595 bp one-end closed DNA with a 12 nt 5' ssDNA tail at low force in 1 µM RecA, 50 mM KCl, 10 mM MgCl_2_, 1 mM ATP, 1x ATP regeneration system, pH 7.4, and 24°C.** RecA polymerization started during introduction of RecA solution, and it fully polymerized after solution exchange finished. The solution exchange was slow to maintain < 10 pN in the whole process.
(PNG)

Methods S1 (PDF)

## References

[1] Kowalczykowski SC, Dixon DA, Eggleston AK, Lauder SD, Rehrauer WM (1994) Biochemistry of homologous recombination in Escherichia coli. Microbiol Rev 58: 401–465. 7968921 10.1128/mr.58.3.401-465.1994 PMC372975

[2] Cox MM (2007) Regulation of bacterial RecA protein function. Crit Rev Biochem Mol Biol 42: 41–63. 17364684 10.1080/10409230701260258

[3] Cox JM, Tsodikov OV, Cox MM (2005) Organized unidirectional waves of ATP hydrolysis within a RecA filament. PLoS Biol 3: e52. 15719060 10.1371/journal.pbio.0030052 PMC546331

[4] Bell JC, Plank JL, Dombrowski CC, Kowalczykowski SC (2012) Direct imaging of RecA nucleation and growth on single molecules of SSB-coated ssDNA. Nature 491: 274–U144. 23103864 10.1038/nature11598 PMC4112059

[5] Joo C, McKinney SA, Nakamura M, Rasnik I, Myong S, et al (2006) Real-time observation of RecA filament dynamics with single monomer resolution. Cell 126: 515–527. 16901785 10.1016/j.cell.2006.06.042

[6] Mani A, Braslavsky I, Arbel-Goren R, Stavans J (2010) Caught in the act: the lifetime of synaptic intermediates during the search for homology on DNA. Nucleic Acids Res 38: 2036–2043. 20044347 10.1093/nar/gkp1177 PMC2847238

[7] Fu H, Le S, Chen H, Muniyappa K, Yan J (2013) Force and ATP hydrolysis dependent regulation of RecA nucleoprotein filament by single-stranded DNA binding protein. Nucleic Acids Res 41: 924–932. 23221642 10.1093/nar/gks1162 PMC3553936

[8] Galletto R, Amitani I, Baskin RJ, Kowalczykowski SC (2006) Direct observation of individual RecA filaments assembling on single DNA molecules. Nature 443: 875–878. 16988658 10.1038/nature05197

[9] Cox JM, Abbott SN, Chitteni-Pattu S, Inman RB, Cox MM (2006) Complementation of one RecA protein point mutation by another. Evidence for trans catalysis of ATP hydrolysis. J Biol Chem 281: 12968–12975. 16527806 10.1074/jbc.M513736200

[10] Pugh BF, Cox MM (1988) General Mechanism for Reca Protein-Binding to Duplex DNA. J Mol Biol 203: 479–493. 3058986 10.1016/0022-2836(88)90014-9

[11] Shivashankar GV, Feingold M, Krichevsky O, Libchaber A (1999) RecA polymerization on double-stranded DNA by using single-molecule manipulation: the role of ATP hydrolysis. Proc Natl Acad Sci U S A 96: 7916–7921. 10393922 10.1073/pnas.96.14.7916 PMC22162

[12] Hegner M, Smith SB, Bustamante C (1999) Polymerization and mechanical properties of single RecA-DNA filaments. Proc Natl Acad Sci U S A 96: 10109–10114. 10468570 10.1073/pnas.96.18.10109 PMC17850

[13] Leger JF, Robert J, Bourdieu L, Chatenay D, Marko JF (1998) RecA binding to a single double-stranded DNA molecule: a possible role of DNA conformational fluctuations. Proc Natl Acad Sci U S A 95: 12295–12299. 9770480 10.1073/pnas.95.21.12295 PMC22825

[14] Feinstein E, Danilowicz C, Conover A, Gunaratne R, Kleckner N, et al (2011) Single-molecule studies of the stringency factors and rates governing the polymerization of RecA on double-stranded DNA. Nucleic Acids Res 39: 3781–3791. 21245047 10.1093/nar/gkr013 PMC3089484

[15] van der Heijden T, van Noort J, van Leest H, Kanaar R, Wyman C, et al (2005) Torque-limited RecA polymerization on dsDNA. Nucleic Acids Res 33: 2099–2105. 15824062 10.1093/nar/gki512 PMC1075924

[16] Sagi D, Tlusty T, Stavans J (2006) High fidelity of RecA-catalyzed recombination: a watchdog of genetic diversity. Nucleic Acids Res 34: 5021–5031. 16990254 10.1093/nar/gkl586 PMC1636419

[17] Fu H, Chen H, Marko JF, Yan J (2010) Two distinct overstretched DNA states. Nucleic Acids Res 38: 5594–5600. 20435680 10.1093/nar/gkq309 PMC2938222

[18] Fu H, Chen H, Zhang X, Qu Y, Marko JF, et al (2011) Transition dynamics and selection of the distinct S-DNA and strand unpeeling modes of double helix overstretching. Nucleic Acids Res 39: 3473–3481. 21177651 10.1093/nar/gkq1278 PMC3082884

[19] Zhang X, Chen H, Fu H, Doyle PS, Yan J (2012) Two distinct overstretched DNA structures revealed by single-molecule thermodynamics measurements. Proc Natl Acad Sci U S A 109: 8103–8108. 22532662 10.1073/pnas.1109824109 PMC3361402

[20] Bosaeus N, El-Sagheer AH, Brown T, Smith SB, Akerman B, et al (2012) Tension induces a base-paired overstretched DNA conformation. Proc Natl Acad Sci U S A 109: 15179–15184. 22949705 10.1073/pnas.1213172109 PMC3458322

[21] Vaze MB, Muniyappa K (1999) RecA protein of Mycobacterium tuberculosis possesses pH-dependent homologous DNA pairing and strand exchange activities: implications for allele exchange in mycobacteria. Biochemistry 38: 3175–3186. 10074373 10.1021/bi9819125

[22] Gosse C, Croquette V (2002) Magnetic tweezers: micromanipulation and force measurement at the molecular level. Biophys J 82: 3314–3329. 12023254 10.1016/S0006-3495(02)75672-5 PMC1302119

[23] Chen H, Fu H, Zhu X, Cong P, Nakamura F, et al (2011) Improved high-force magnetic tweezers for stretching and refolding of proteins and short DNA. Biophys J 100: 517–523. 21244848 10.1016/j.bpj.2010.12.3700 PMC3325116

[24] Yan J, Skoko D, Marko JF (2004) Near-field-magnetic-tweezer manipulation of single DNA molecules. Phys Rev E 70: 011905. 10.1103/PhysRevE.70.011905 15324086

[25] Cluzel P, Lebrun A, Heller C, Lavery R, Viovy JL, et al (1996) DNA: an extensible molecule. Science 271: 792–794. 8628993 10.1126/science.271.5250.792

[26] Smith SB, Cui Y, Bustamante C (1996) Overstretching B-DNA: the elastic response of individual double-stranded and single-stranded DNA molecules. Science 271: 795–799. 8628994 10.1126/science.271.5250.795

[27] Zhang X, Chen H, Le S, Rouzina I, Doyle PS, et al (2013) Revealing the competition between peeled ssDNA, melting bubbles, and S-DNA during DNA overstretching by single-molecule calorimetry. Proc Natl Acad Sci U S A 110: 3865–3870. 23431154 10.1073/pnas.1213740110 PMC3593865

[28] Cocco S, Yan J, Leger JF, Chatenay D, Marko JF (2004) Overstretching and force-driven strand separation of double-helix DNA. Phys Rev E 70: 011910. 10.1103/PhysRevE.70.011910 15324091

[29] Hsu HF, Ngo KV, Chitteni-Pattu S, Cox MM, Li HW (2011) Investigating Deinococcus radiodurans RecA protein filament formation on double-stranded DNA by a real-time single-molecule approach. Biochemistry 50: 8270–8280. 21853996 10.1021/bi200423t PMC3183162

[30] Fulconis R, Bancaud A, Allemand JF, Croquette V, Dutreix M, et al (2004) Twisting and untwisting a single DNA molecule covered by RecA protein. Biophys J 87: 2552–2563. 15454450 10.1529/biophysj.104.043059 PMC1304674

[31] Mazin AV, Kowalczykowski SC (1996) The specificity of the secondary DNA binding site of RecA protein defines its role in DNA strand exchange. Proc Natl Acad Sci U S A 93: 10673–10678. 8855238 10.1073/pnas.93.20.10673 PMC38213

