# Supplementary figures and images for: Dynamics and Regulation of RecA Polymerization and De-Polymerization on Double-Stranded DNA
Source: PLoS One. 2013 Jun 18;8(6):e66712. doi: 10.1371/journal.pone.0066712 (PMC3688958; doi:10.1371/journal.pone.0066712)

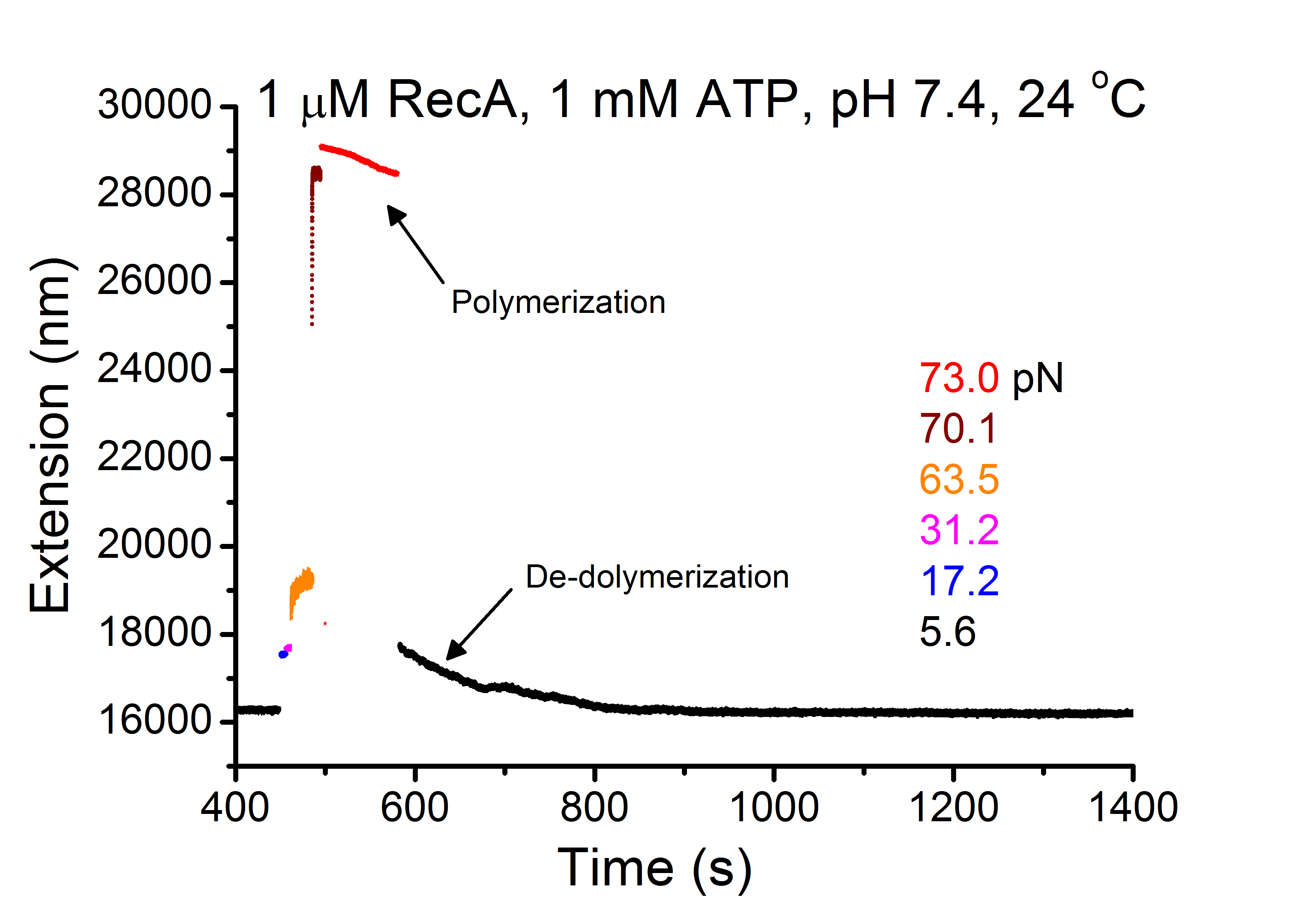

Supplement: Figure S1 — Time trace of RecA polymerization and de-polymerization on a λ-DNA in 1 µM RecA, 50 mM KCl, 10 mM MgCl2, 1 mM ATP, 1X ATP regeneration system, pH 7.4, and 24 °C at different forces indicated by different colors. Progressive polymerization was observed at ∼73 pN after DNA overstretching indicated by shortening in DNA extension (Red), while de-polymerization was observed when force was decreased to ∼5.6 pN (black). (PNG) [file pone.0066712.s001.png]

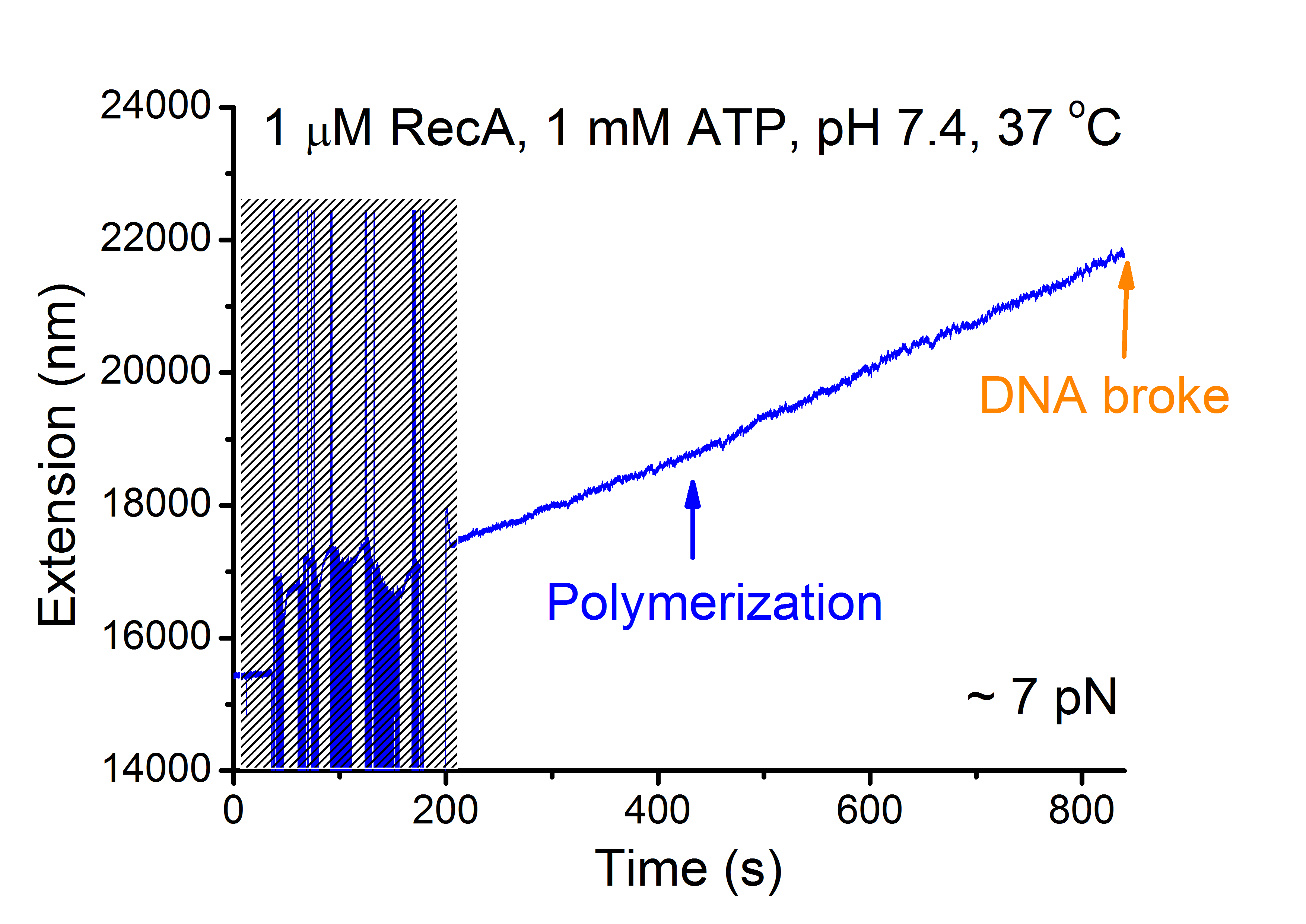

Supplement: Figure S2 — Time trace of spontaneous RecA polymerizing on a λ-DNA in 1 µM RecA, 50 mM KCl, 10 mM MgCl2, 1 mM ATP, 1X ATP regeneration system, pH 7.4, and 37°C, at a force of ∼ 7 pN. Progressive polymerization was observed (blue arrow) before the DNA was broken after 800 second (orange arrow). The noisy data in the shadowed areas were recorded during buffer exchanging. (PNG) [file pone.0066712.s002.png]

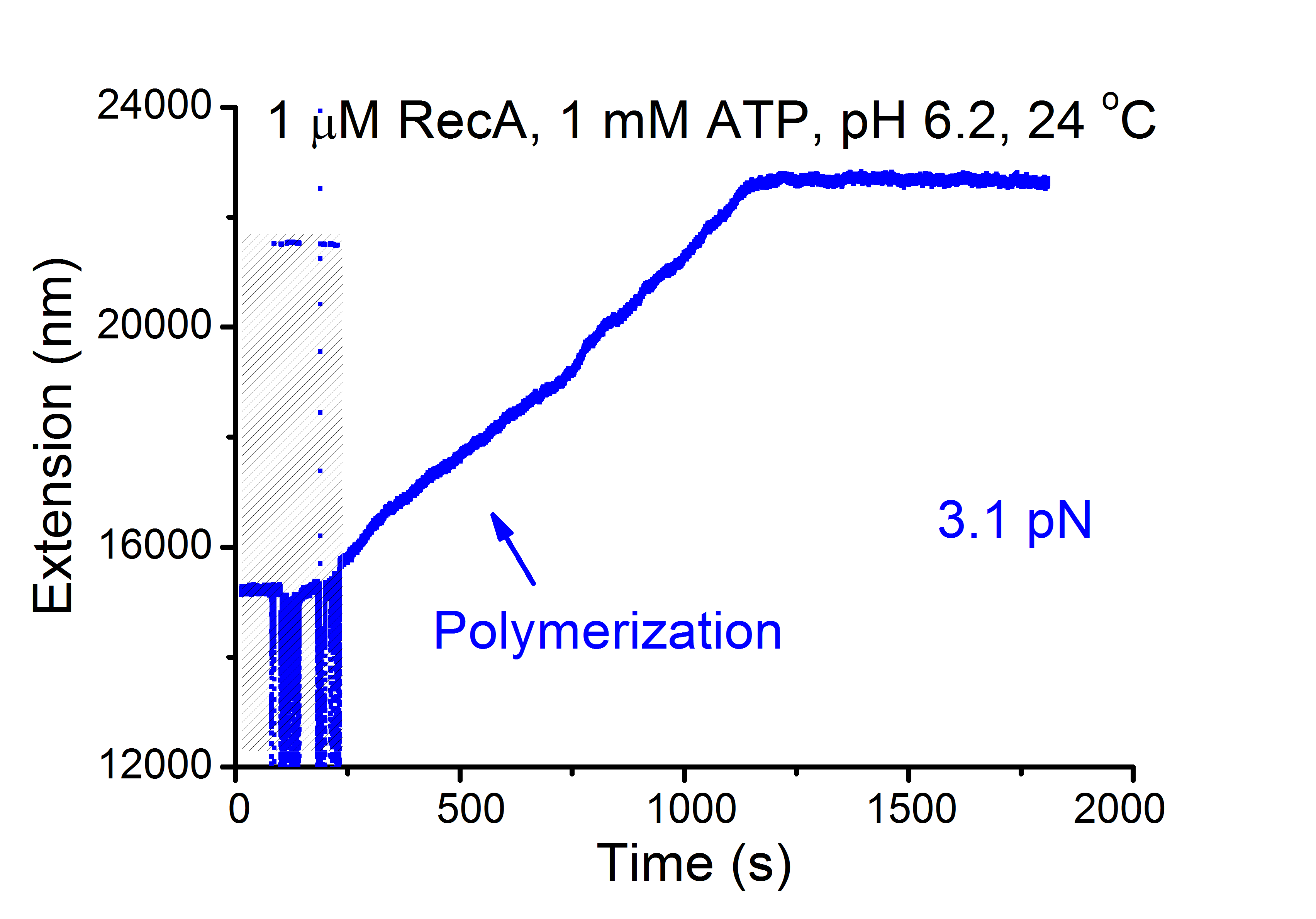

Supplement: Figure S3 — Time trace of spontaneous RecA polymerizing on a λ-DNA in 1 µM RecA, 50 mM KCl, 10 mM MgCl2, 1 mM ATP, 1X ATP regeneration system, pH 6.2, and 24°C, at a force of ∼ 3.1 pN. The noisy data in the shadowed areas were recorded during buffer exchanging. (PNG) [file pone.0066712.s003.png]

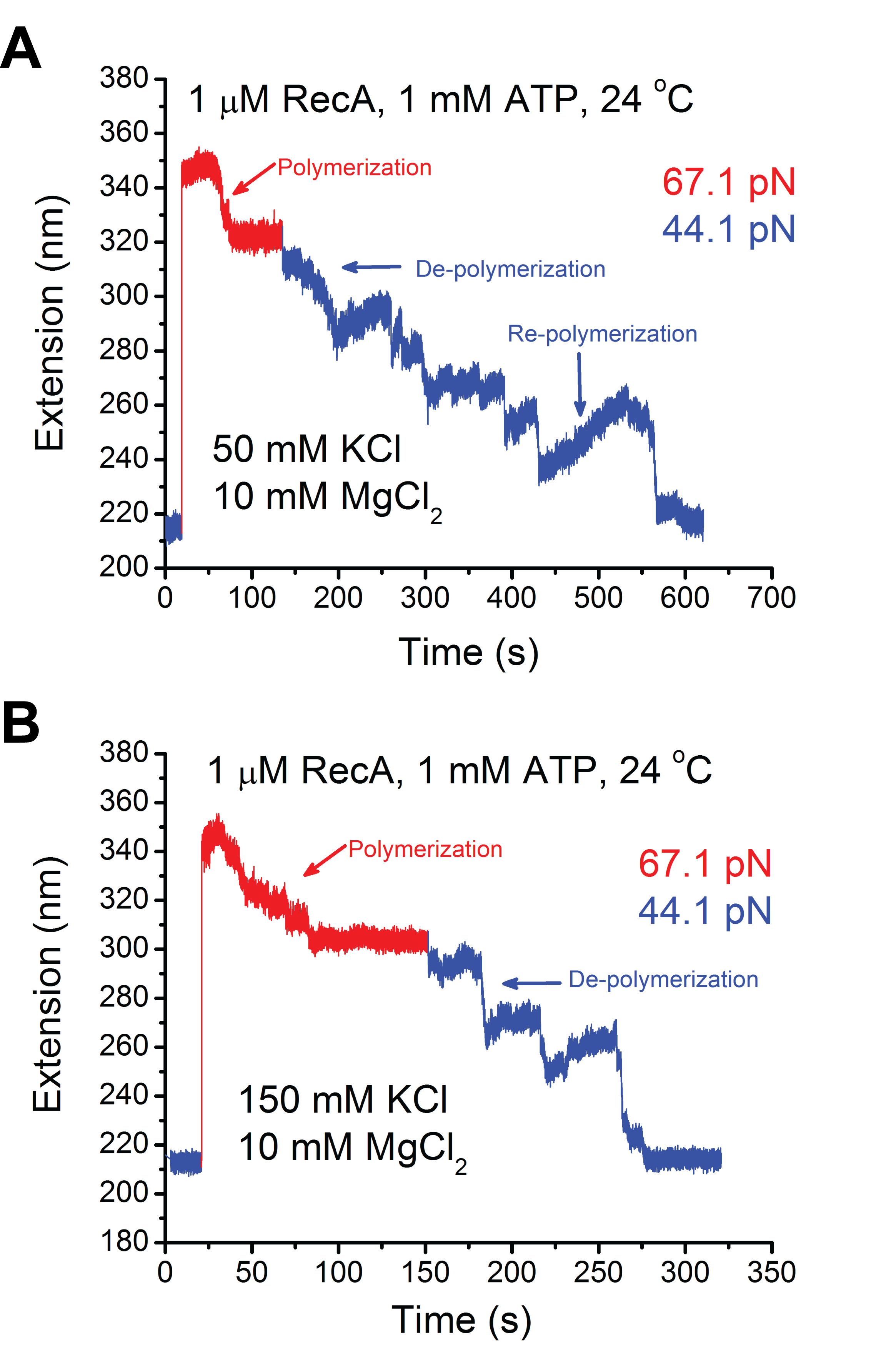

Supplement: Figure S4 — Effects of KCl concentration on the dynamics of RecA filaments. (A–B) Time traces of polymerization and de-polymerization of RecA filament on a 595 bp dsDNA in 1 µM RecA, 10 mM MgCl2, 1 mM ATP, 1X ATP regeneration system, pH 7.4, and 24°C, with 50 mM KCl (A) first then 150 mM KCl (B). In both A and B, progressive RecA polymerization was observed after the force was jumped from 44.1 pN to 67.1 pN (red). Different dynamics of de-polymerization and polymerization of 50 mM KCl and 150 mM KCl were observed when force was jumped back to 44.1 pN (blue). (PNG) [file pone.0066712.s004.png]

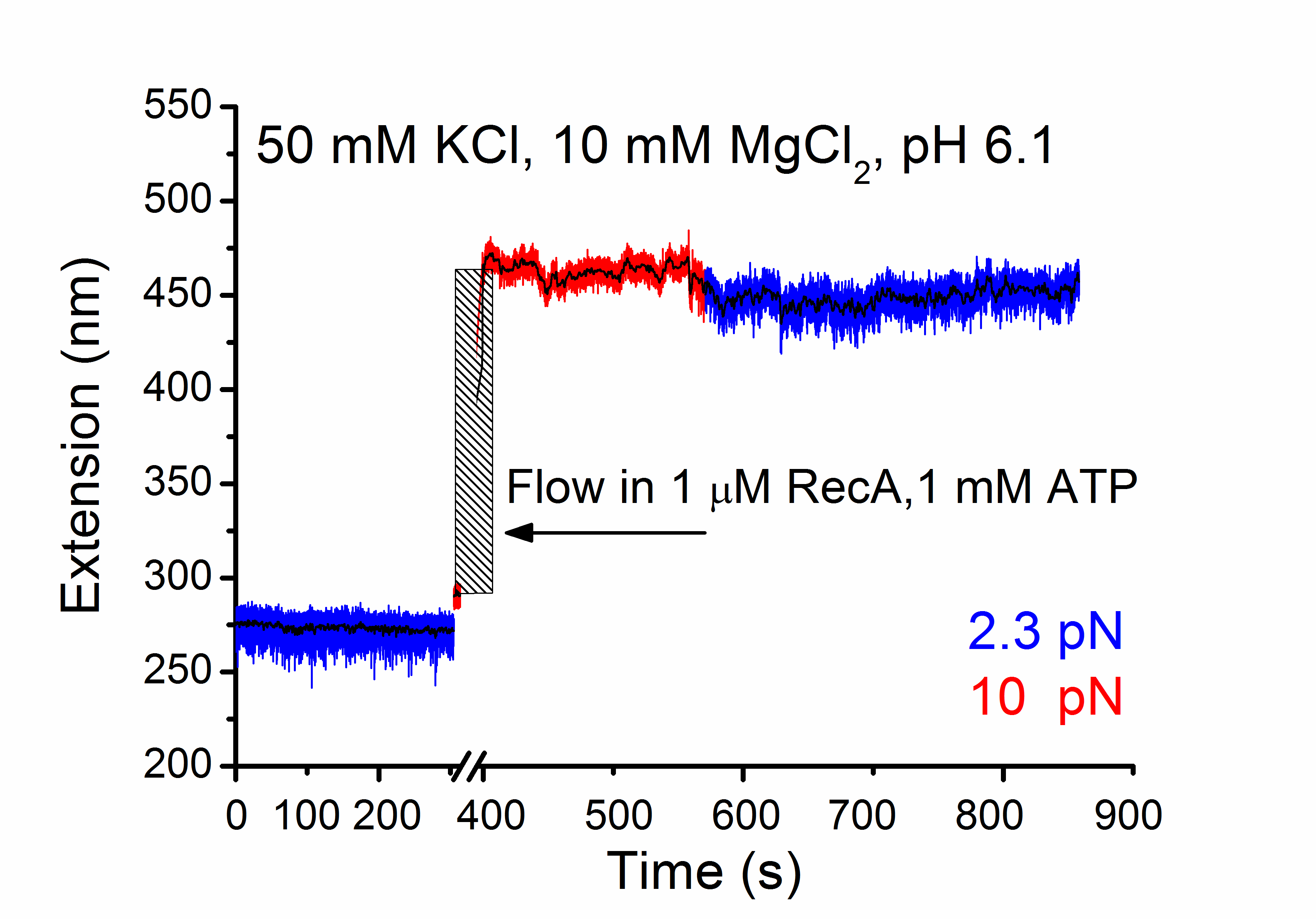

Supplement: Figure S5 — Time trace of spontaneous RecA polymerizing on a 595 bp DNA in 1 µM RecA, 50 mM KCl, 10 mM MgCl2, 1 mM ATP, 1X ATP regeneration system, pH 6.1, and 24°C, at a force of ∼10 pN and stable RecA filament at < 3 pN. The noisy data in the shadowed areas were recorded during buffer exchanging. (PNG) [file pone.0066712.s005.png]

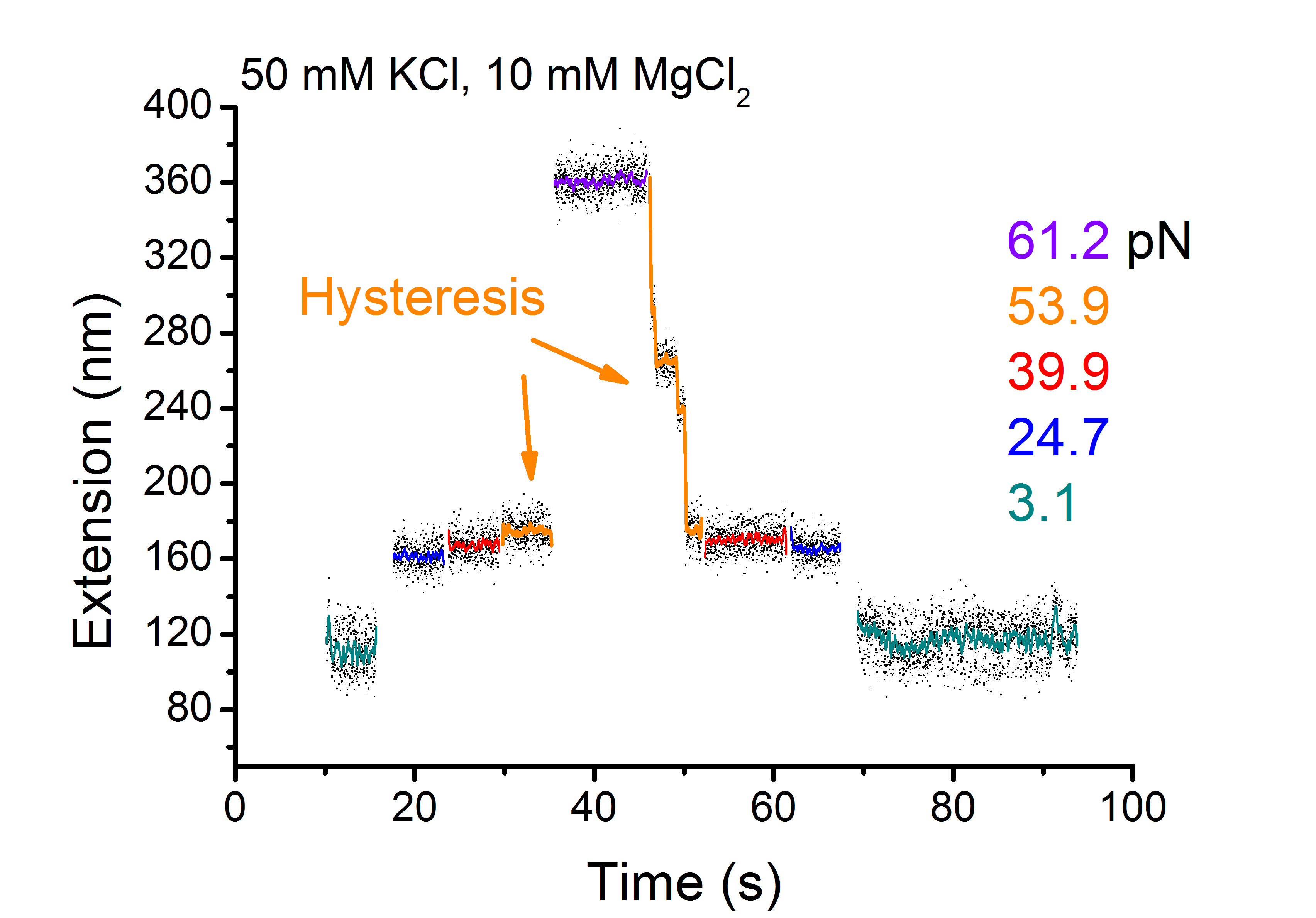

Supplement: Figure S6 — Extension hysteresis between force-decrease scan and force increase scan during the strand peeling transition. Time trace of force-increase scan and force-decrease scan of a 595 bp DNA in 50 mM KCl, 10 mM MgCl2, 1 mM ATP, 1X ATP regeneration system, pH 7.4, and 24°C, at different forces indicated by different colors. The hysteresis indicated by different extensions at 53.9 pN (orange) between the two force-scans suggests that the DNA went through a strand peeling transition during the force-increase scan. (PNG) [file pone.0066712.s006.png]

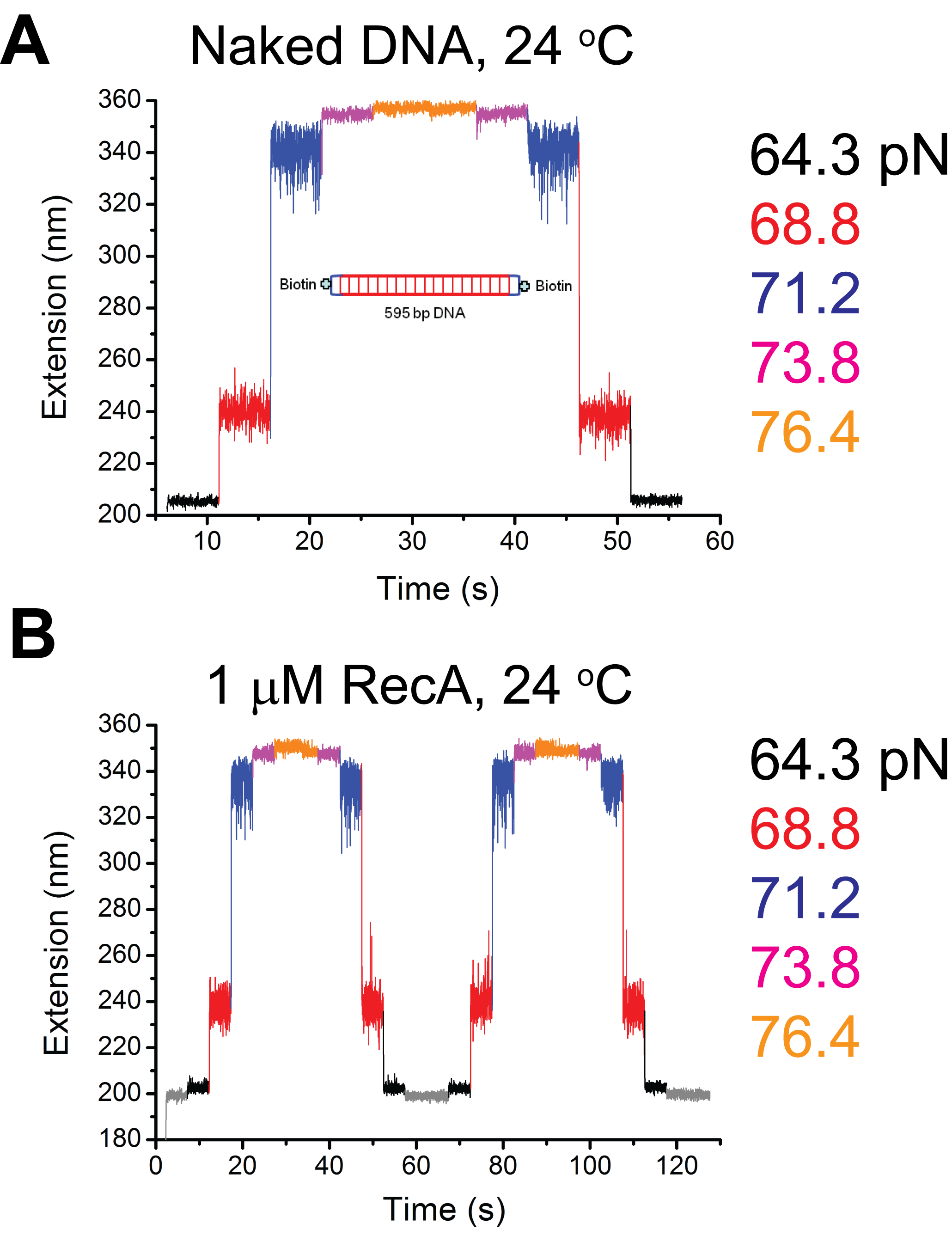

Supplement: Figure S7 — Time traces of an ∼600 bp two-ends-closed DNA in 50 mM KCl, 10 mM MgCl2, pH 7.4, and 24°C, without RecA (A) or with 1 µM RecA,1 mM ATP, 1x ATP regeneration system (B). The DNA overstretching transitions in both A and B are completely reversible and without hysteresis, indicated by the same extensions recorded at the same forces during the force-increase and force-decrease scans. These results indicate that no RecA filaments formed on the S-DNA produced during B-to-S transition within the experimental time scale. Inset shows a sketch of the end-closed DNA. (PNG) [file pone.0066712.s007.png]

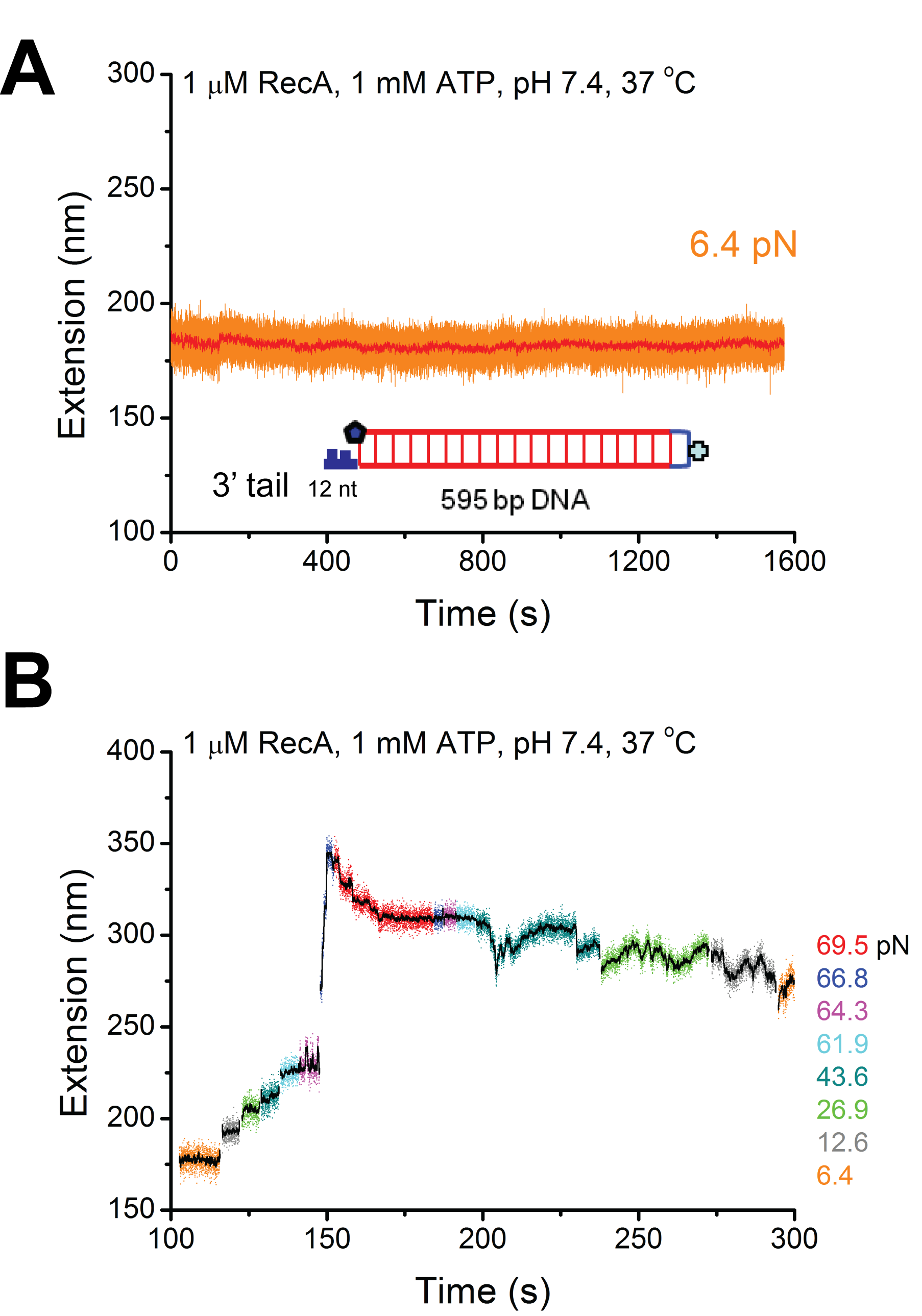

Supplement: Figure S8 — Time traces of the extension of a 595 bp DNA with a 12 nt 3' ssDNA tail and another end sealed. (A) Time traces of the extension of the DNA in 1 µM RecA, 50 mM KCl, 10 mM MgCl2, 1 mM ATP, 1x ATP regeneration system, pH 7.4, and 37°C. Within the experimental time scale of 1600 seconds, the DNA extension remained at the B-DNA extension, indicating that 3' ssDNA overhang did not promote RecA filament formation at low force. (B) Time trace of the same DNA in the same solution and temperature condition, when the force was subsequently increased to > 60 pN where DNA overstretching transition occurred, the RecA polymerization immediately started and the resulting RecA filament was stable. When the force was reduced to 6.4 pN, the DNA extension was still ∼120 nm longer than the B-DNA before RecA polymerization, indicating a stable RecA filament at low force. (PNG) [file pone.0066712.s008.png]

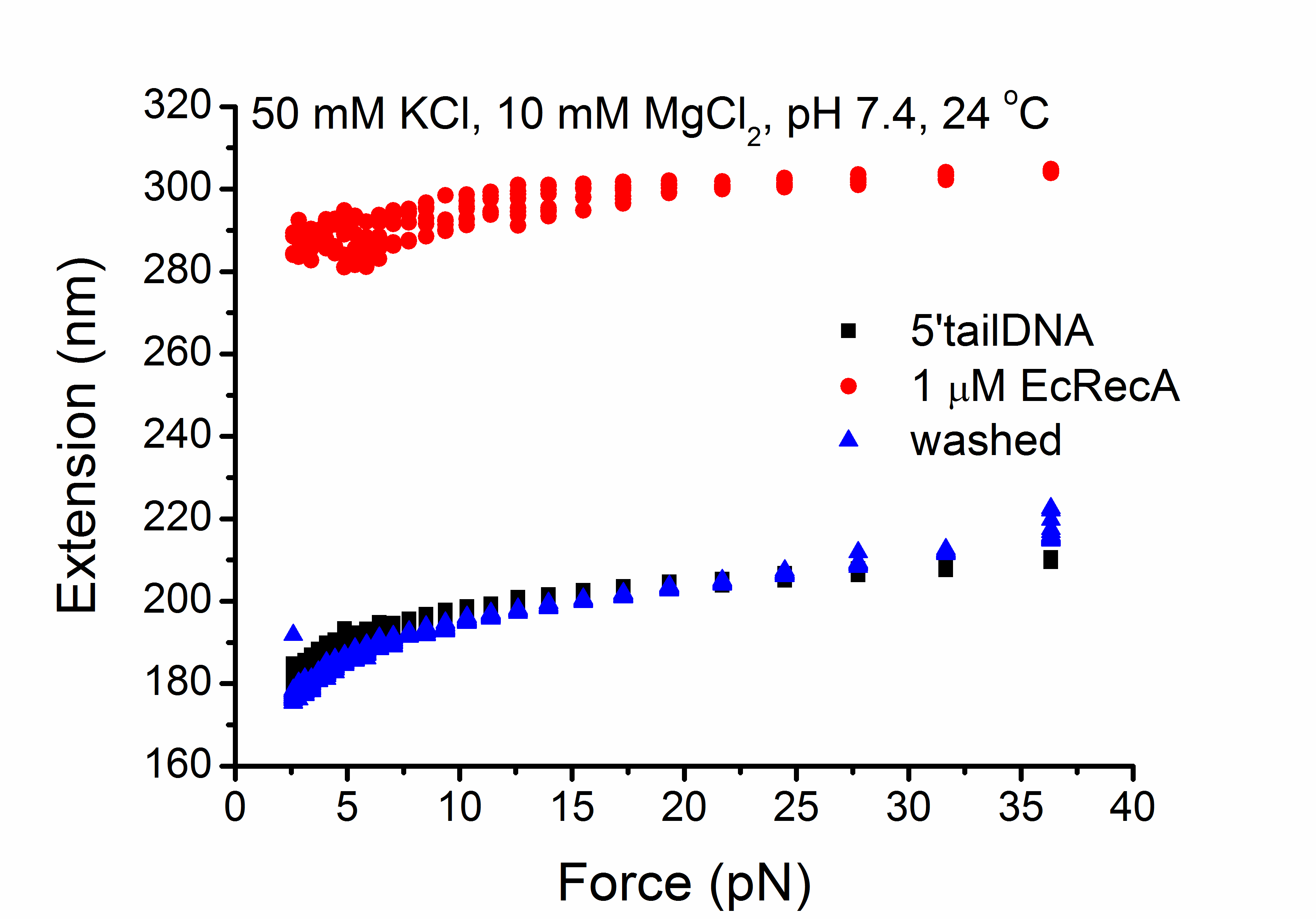

Supplement: Figure S9 — Force responses of a 595 bp one-end closed DNA with a 12 nt 5' ssDNA (Black), and the same DNA with RecA filament formed (Red). The extension of the DNA formed with RecA filament is about 50% longer than that of naked DNA before RecA was introduced. After remove the RecA by exchanging to pure buffered solution, the RecA filament de-polymerized and resulting DNA extension (blue) overlaps with naked DNA extension. The DNA used in this experiment is the same as that used in Figure 5B in the main text. (PNG) [file pone.0066712.s009.png]

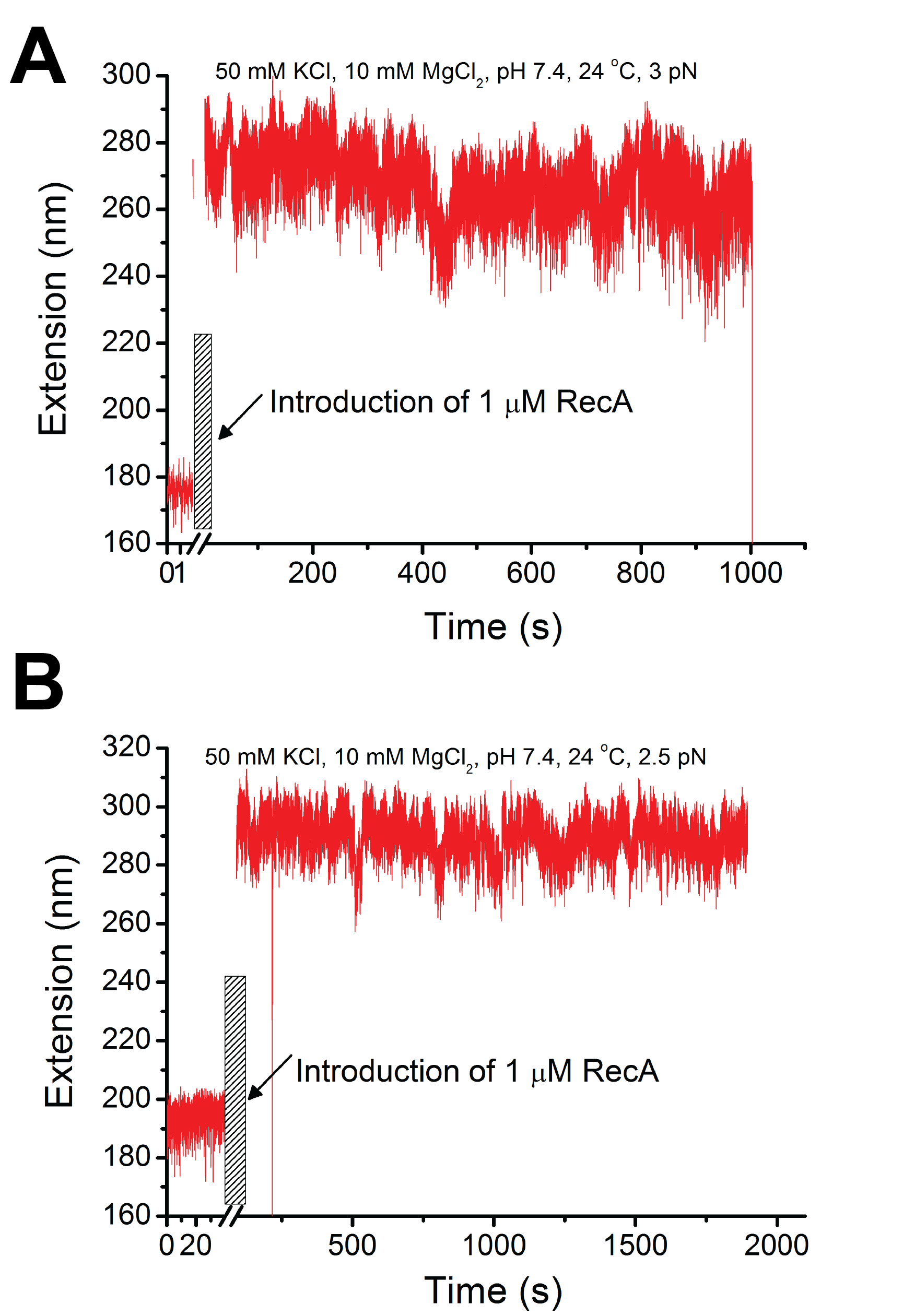

Supplement: Figure S10 — Spontaneous RecA polymerization on two independent 595 bp one-end closed DNA with a 12 nt 5' ssDNA tail at low force in 1 µM RecA, 50 mM KCl, 10 mM MgCl2, 1 mM ATP, 1x ATP regeneration system, pH 7.4, and 24°C. RecA polymerization started during introduction of RecA solution, and it fully polymerized after solution exchange finished. The solution exchange was slow to maintain < 10 pN in the whole process. (PNG) [file pone.0066712.s010.png]
