# Supplementary material for: Dynamics and Regulation of RecA Polymerization and De-Polymerization on Double-Stranded DNA
Source: PLoS One. 2013 Jun 18;8(6):e66712. doi: 10.1371/journal.pone.0066712 (PMC3688958; doi:10.1371/journal.pone.0066712)
Supplement: Methods S1 — (PDF) [file pone.0066712.s011.pdf]

SUPPLEMENTARY INFORMATION
Dynamics and Regulation of RecA polymerization and de-polymerization on double-stranded DNA

Hongxia Fu,,* Shimin Le,,* K Muniyappa, and Jie Yan,†
(Dated: May 16, 2013)

Methods-S1

Text-DNA constructs, labelling, and ATP regeneration system.—Using DreamTaq DNA polymerase (Fermentas), the 576 bp DNA construct was generated by PCR from bacteriophage λ-DNA. The following primers were used to generate the sequence from $992-1552$ bp on $\lambda-$DNA in PCR.
5'[Thiol]ATTATACTCGAGAGCATAAGCAGCGCAA CA3' and 5'ATTATAGAATTCATGACGCAGGCAT TAT-GCT3'
The 595 bpDNA is from ligation of the 576 bp DNA with following oligos: 5-[phosphate]cggcatggtatAGATTT[Biotin]TTATCTATAC CATGCCGCTC-3'
The thiol-λ-biotin DNA is constructed by ligating the two opposite ends of the $\lambda-$DNA (NEB) with thiol-labelled and biotin-labelled oligos.
The DNA is tethered between a streptavidin coated paramagnetic bead (Dynalbeads M-280 Streptavidin, Invitrogen) and a sulfo-SMCC-coated coverglass. To prepare the sulfo-SMCC-coated coverglass, the glass surface was cleaned and sinalized with (3-Aminopropyl)triethoxysilane (APTES) (Sigma). Then, it was incubated with sulfo-SMCC (Sigma) in 1x PBS buffer for $\sim$30 minutes. Right after the sulfo-SMCC coverglass was prepared, the labeled DNA solution in 1x PBS was introduced and incubated with the surface for $\sim$30 minutes. Then, the channel was blocked with 2% BSA in 1xPBS for 1 hour before the streptavidin-coated paramagnetic beads were introduced and incubated for 10 minutes to form tethers. A single dsDNA tether was identified by the unique DNA overstretching transition at $\sim$ 65 pN with$\sim$ $1.7-$fold elongation in extension (1,2).
ATP regeneration system (10x) recipe: 20 mM phosphoenol (sigma P0564), 100 unit/ml pyruvate kinase (sigma 9136), 10 mM KCL. The pH was adjusted to 7.5.

Force-responses and force-calibration of short dsDNA.—The force-extension curve of dsDNA has been extensively measured (3,4), and can be fitted by the extensible worm-like-chain model at larger force range (2,4), as described by the following equation:

$$E_{\mathrm{ds,model}}(f) = L(1 - \mathrm{sqrt}(k_BT/(4Af) + f/f_s) \quad (1)$$

where $L = N \times 0.34$ nm is the contour length of dsDNA of N base pairs, $A = 50$ nm is the persistence length of B-DNA, and $f_s = 1400$ pN is the force constant describing the backbone stretching elasticity. The force $f(d)$ depends on the distance d between the bead and the pair of magnets through a double exponential decay function (5):

$$F(d) = C \times (\alpha_1 \exp(-d/\gamma) + \alpha_2 \exp(-d/\gamma_2)) \quad (2)$$

where $\alpha_1, \alpha_2, \gamma_1, \gamma_2$ are fitting parameters that describe the magnetic field. These parameters are constants for given magnets, and were determined in the same way as described in our previous publication (5). C is a bead-dependent parameter which is related to the maximal magnetization of the bead. At a given value of d, in addition to the force applied by the magnetic field, there is also a torque due to the alignment of the bead along the magnetic field, which is balanced by the torque applied from tension on DNA. A change in d will change both the force and re-balance of torque which will cause rotation of the bead (see details in Supplementary Materials in (6)). For short DNA tether, the bead rotation will also change the height of bead which cannot be differentiated from real extension change of the DNA (6). Therefore, we only focus on the extension changes at the same force in different cycles of force scans.

Distinctive extension dynamics of unpeeling and S-DNA transition.—The differential extension dynamics of unpeeling and S-DNA transition, as well as the selection of the two transition modes in various experimental conditions, have been studied in detail (7,8,9). The extension hysteresis between force-decrease scan and force increase scan in supplementary Figure S4 and Figure 2A in reference 8 are results from the strand peeling of

the DNA during the force-increase scan, leading to partially peeled two ssDNA strands at higher force. In the force-decrease scan, the two partially peeled ssDNA strands re-annealed gradually. The overlapping extensions from the force-decrease scan and force-increase scan in Figure 4A-C in the main text and Figure 2B-C in reference 8 indicate the B-to-S transion (7,8,9).

---

* Joint First Authors
† Correspondence should be addressed to: phyyj@nus.edu.sg

1. Smith, S.B., Cui, Y. and Bustamante, C. (1996) Overstretching B-DNA: the elastic response of individual double-stranded and single-stranded DNA molecules. Science, 271, 795-799.
2. Cluzel, P., Lebrun, A., Heller, C., Lavery, R., Viovy, J.L., Chatenay, D. and Caron, F. (1996) DNA: an extensible molecule. Science, 271, 792-794.
3. Smith, S.B., Finzi, L. and Bustamante, C. (1992) Direct mechanical measurements of the elasticity of single DNA molecules by using magnetic beads. Science, 258, 1122-1126.
4. Marko, J.F. and Siggia, E.D. (1995) Stretching DNA. Macromolecules, 28, 12.
5. Chen, H., Fu, H., Zhu, X., Cong, P., Nakamura, F. and Yan, J. (2011) Improved high-force magnetic tweezers for stretching and refolding of proteins and short DNA. Biophys J, 100, 517-523.
6. Chen, H., Zhu, X., Cong, P., Sheetz, M.P., Nakamura, F. and Yan, J. (2011) Differential mechanical stability of filamin a rod segments. Biophys J, 101, 1231-1237.
7.Fu, H., Chen, H., Marko, J.F. and Yan, J. (2010) Two distinct overstretched DNA states. Nucleic acids research, 38, 5594-5600.
8. Fu, H., Chen, H., Zhang, X., Qu, Y., Marko, J.F. and Yan, J. (2011) Transition dynamics and selection of the distinct S-DNA and strand unpeeling modes of double helix overstretching. Nucleic acids research, 39, 3473-3481.
9. Zhang, X., Chen, H., Fu, H., Doyle, P.S. and Yan, J. (2012) Two distinct overstretched DNA structures revealed by single-molecule thermodynamics measurements. Proc Natl Acad Sci U S A, 109, 8103-8108.
